# Diversity and distribution of bacterial DNA polymerases

**DOI:** 10.1093/nar/gkag133

**Published:** 2026-02-23

**Authors:** Kęstutis Timinskas, Darius Kazlauskas, Albertas Timinskas, Česlovas Venclovas

**Affiliations:** Institute of Biotechnology, Life Sciences Center, Vilnius University, Saulėtekio 7, Vilnius, LT-10257, Lithuania; Institute of Biotechnology, Life Sciences Center, Vilnius University, Saulėtekio 7, Vilnius, LT-10257, Lithuania; Institute of Biotechnology, Life Sciences Center, Vilnius University, Saulėtekio 7, Vilnius, LT-10257, Lithuania; Institute of Biotechnology, Life Sciences Center, Vilnius University, Saulėtekio 7, Vilnius, LT-10257, Lithuania

## Abstract

DNA polymerases are key players in DNA replication, repair, and maintenance. However, the overall abundance, diversity, and distribution of bacterial DNA polymerases have not been systematically explored. To close this knowledge gap, we computationally identified and characterized DNA polymerases and their homologs from A, B, C, X, and Y families in over 3000 representative bacterial species with complete genomes. We found that Y-family is the most abundant, followed by C and A families, whereas B and X families are rare. All species have replicative C-family polymerases, 96% have A-family polymerases, and 88% have Y-family members. In each family, we identified and annotated distinct groups, proofreading nucleases, and interaction motifs. Based on conserved associations for DnaE2 and Y-family groups, we identified 11 types of putative multimeric error-prone DNA polymerases supported by AlphaFold modeling. Approximately 90% of the complexes belong to four major types, exemplified by *Meiothermus silvanus* PolY–RecA complex, *Mycobacterium tuberculosis* ImuA–ImuB–DnaE2, *Escherichia coli* Pol V (UmuC–UmuD′_2_–RecA), and *Bacillus subtilis* YqjW–YqjX–RecA. We found that distribution patterns of distinct polymerase groups and multimeric complexes are shaped by bacterial lineages, replication-system types, and environmental factors. Our results thus provide a comprehensive picture of DNA polymerase diversity and distribution across the bacterial domain.

## Graphical Abstract

**Graphical Abstract:**
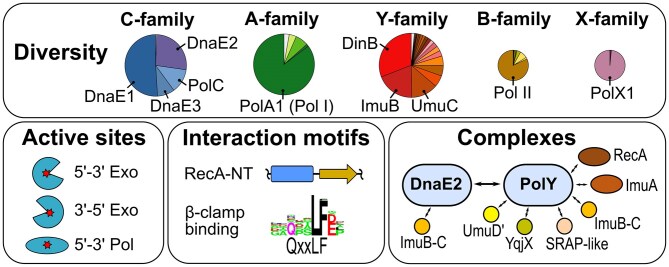

## Introduction

DNA polymerases play essential roles in replication, maintenance, and repair of DNA. Based on phylogenetic relationships, they are classified into A, B, C, D, X, Y, and reverse transcriptase (RT) families [1, 2]. The first six famgilies represent DNA-dependent DNA polymerases, whereas RTs are RNA-dependent DNA polymerases. Strikingly, despite the common biochemical function, the catalytic cores of DNA polymerase families represent three evolutionarily unrelated structural folds. Catalytic domains of the A, B, Y, and RT family DNA polymerases structurally resemble the *Escherichia coli* Pol I Klenow-fold, also known as the “ferredoxin-like” fold [3]. Families C and X feature the Polβ-like fold, often referred to as the “nucleotidyltransferase” fold, whereas the catalytic subunit of D-family polymerases has the “double barrel” fold also found in RNA polymerases [2, 3]. Out of seven DNA polymerase families, bacteria lack D-family, members of which so far have been found only in archaea [1]. Although homologs of another family, RT, are usually thought of as eukaryotic and viral enzymes, they are also present in bacteria, but mostly as components of retroelements and phage defense systems [4, 5]. Consequently, out of all DNA polymerase families, A, B, C, X, and Y are the most relevant for duplication and maintenance of genomic DNA in bacteria.

The key bacterial DNA polymerases performing replication of chromosomal DNA belong to the C-family. This family is divided into four major groups, DnaE1, DnaE2, DnaE3, and PolC [6, 7]. DnaE1, DnaE3, and PolC represent distinct types of the essential catalytic α-subunit of the DNA polymerase III holoenzyme. DnaE1, typified by *E. coli* Pol III α-subunit, is sufficient for the replication of both leading and lagging strands [8]. In *Bacillus subtilis* and some other bacteria, DNA replication involves two essential C-family enzymes, PolC and DnaE3 [9–11]. There is also another combination of replicative polymerases, PolC and DnaE1, found mainly in Clostridia and Fusobacteria [7]. To increase replication accuracy, Pol III α-subunits utilize 3′–5′ proofreading exonucleases of two different types. One type is represented by the intrinsic polymerase and histidinol phosphatase (PHP) domain, which is universally present in C-family polymerases but is frequently inactivated [7, 12–15]. Another type is represented by the unrelated DnaQ exonuclease with the “DEDDh” active site motif [16]. DnaQ may exist as a domain within the Pol III α-subunit, or as a separate protein encoded by the *dnaQ* gene exemplified by the *E. coli* Pol III ε-subunit [17]. The fourth C-family group, DnaE2, represents non-essential DNA polymerases involved in error-prone DNA repair. In several bacteria DnaE2 was found to function together with ImuA, a homolog of the RecA recombinase, and ImuB, a catalytically inactive PolY-like protein [18–20].

Bacterial DNA polymerases of A-family are typified by *E. coli* Pol I, the very first DNA polymerase discovered [21]. In addition to polymerase activity, Pol I has two distinct nuclease activities residing in separate domains upstream of the polymerase core. The N-terminal domain with 5′–3′ exonuclease and 5′-flap endonuclease activities, belongs to the flap endonuclease (FEN) family [22, 23]. The second domain has the 3′–5′ proofreading exonuclease activity. As in the case of C-family, it belongs to the DnaQ-like exonucleases, but with the “DEDDy” active site motif instead of “DEDDh” [16, 24, 25]. Removal of the N-terminal 5′–3′ exonuclease domain produces the so-called Klenow fragment, a Pol I derivative containing only DNA polymerase and 3′–5′ exonuclease activities [26]. The primary role of Pol I in DNA replication is to remove RNA primers and fill in the resulting gaps during Okazaki fragment maturation [27]. In addition, Pol I has been implicated in replication termination [28]. The 5′–3′ exonuclease domain is essential for fulfilling both of these functions. Pol I is also known to fill gaps generated during DNA repair and recombination [29]. In addition to prototypical Pol I, a number of other bacterial PolA groups were identified, either as a replacement of canonical Pol I or as accessory polymerases [30, 31].

B-family DNA polymerases are ubiquitous in both cellular organisms and viruses [32]. In eukaryotes and several archaeal lineages, members of B-family function as the essential replicative DNA polymerases [2]. In contrast, bacterial B-family DNA polymerases are non-essential enzymes that play accessory roles. An archetypal bacterial B-family DNA polymerase, *E. coli* Pol II, is a SOS-inducible enzyme that participates in translesion DNA synthesis (TLS) [33, 34]. In addition to DNA polymerase activity, Pol II has the 3′–5′ proofreading exonuclease activity associated with the DnaQ-like domain of the same “DEDDy” variant as in Pol I. This enables Pol II to perform fairly accurate replication of both damaged and undamaged DNA [35]. Overall, Pol II is linked to enhanced survival and evolutionary fitness, pointing to its important physiological role [36]. A comprehensive survey of B-family DNA polymerases revealed that, besides Pol II orthologs, bacteria have two other major groups [32]. One group was shown to be derived from horizontally transferred archaeal PolB2 [32, 37] devoid of the 3′–5′ exonuclease activity and functioning in TLS [38, 39]. Recently, PolB2, with its two partner proteins, was predicted to form a multimeric mutasome complex [40]. Another major group, displaying similarity to the catalytically active N-terminal half of the eukaryotic Pol ε, remains uncharacterized [32].

X-family DNA polymerases were first discovered in eukaryotes, where they are mainly involved in the DNA synthesis step during base excision repair (BER) and double-strand break (DSB) repair [41]. Bacterial PolXs have also been shown to participate in BER and DSB repair [42, 43]. A prototypical bacterial enzyme, *B. subtilis* PolX, has three different catalytic activities: polymerase, 3′–5′ exonuclease and AP-endonuclease [44]. The latter two activities are attributed to the PHP domain, which is not present in eukaryotic PolXs [42, 45]. Recently, a group of noncanonical prokaryotic X-family members was identified. They all lack polymerase activity but retain the PHP domain-associated 3′–5′ exonuclease activity [46].

Y-family comprises DNA polymerases characterized by intrinsic low fidelity and the ability to bypass DNA lesions that usually block polymerases of other families [47]. The most extensively studied bacterial Y-family members are *E. coli* polymerases Pol IV and Pol V. Pol IV corresponds to a single protein chain encoded by the *dinB* gene [48]. In contrast, Pol V corresponds to the UmuD′_2_C heterotrimer, with UmuC representing a Y-family polymerase and UmuD′ an accessory subunit. By itself, UmuD′_2_C has little DNA synthesis activity, and only when bound to RecA and ATP is the fully active Pol V mutasome (Pol V Mut) formed [49, 50]. In *E. coli*, Pol V is the major TLS polymerase, able to traverse many different lesions, while Pol IV is mainly involved in TLS of alkylation damage [51]. *M. tuberculosis* and some other bacteria have a catalytically inactive Y-family homolog, ImuB, which, together with ImuA and DnaE2 (a non-essential C-family polymerase), forms yet another highly mutagenic TLS complex [19, 52]. More recently, computational analysis revealed considerable diversity of bacterial Y-family, exceeding conventional grouping [53]. At the same time, a common sequence motif, homologous to the RecA N-terminal region (RecA-NT) mediating subunit–subunit interactions in the RecA filament, was identified in a number of diverse Y-family groups [53]. This finding suggested that multiple PolYs may interact with RecA or its homologs. Most recently, the importance of the RecA-NT motif in ImuB for binding ImuA, and thereby enabling the DNA damage-induced mutagenesis was demonstrated experimentally [54]. Nonetheless, despite sustained research efforts, so far, no experimentally determined structures are available for any of the multisubunit complexes involving bacterial members of Y-family.

Neither the enzymatic properties nor the cellular function is specific to a given DNA polymerase family. Different members of the same family may perform different functions in the cell, and, *vice versa*, DNA polymerases from different families may perform very similar functions. For a comprehensive understanding of the DNA synthesis capacity in a given bacterial species, one needs to know which DNA polymerases are present in the cell, what their enzymatic activities and cellular functions are, and how these DNA polymerases cooperate with each other. Yet, only in *E. coli* is the complete set of cellular DNA polymerases (Pol I–V) reasonably well characterized. For other bacterial species, characterization of their DNA polymerases is partial at best. Moreover, both the overall abundance and the frequency of DNA polymerases from specific families and subfamilies encoded within individual bacterial genomes have not been explored in a systematic manner.

Here, taking advantage of the availability of a large number of complete genome sequences and corresponding proteomes, we set out to explore the abundance, diversity, and distribution of DNA polymerases in Bacteria. We used sequence, structure, domain composition, and motif analysis to identify distinct groups within each individual family and to infer their biochemical and cellular functions. Furthermore, we identified and computationally characterized a number of novel putative multimeric TLS polymerases. We also investigated the composition of DNA polymerase sets in individual bacterial species and asked whether these sets might reflect general properties of the species and their living environment.

## Materials and methods

### Bacterial genomes, proteomes, and metadata

Complete bacterial genomes were downloaded from the NCBI ftp site (https://ftp.ncbi.nlm.nih.gov/genomes/) [55]. Only complete genomes tagged as “representative” or “reference” were selected for the representative genome set. Bacterial representatives that had low-quality genomes or no associated proteome data were discarded. For bacterial taxonomy, the data provided in the files associated with genomes were used. Data for oxygen usage was obtained from two databases: GOLD [56] and BacDive [57]. Data were matched to the genome set based on genome ID, species name, or class name, whichever was available first. The data from BacDive were assigned first, and the GOLD data were used to further fill in missing values. Oxygen usage classification was reduced into just two classes: aerobes and anaerobes. All bacteria that can use oxygen (including facultative anaerobes) were classified as aerobes; the remaining bacteria were considered anaerobes. For cases where multiple entries were available for the same genome, the more common oxygen usage notation was used, and, in case of a tie, it was treated as if data were not available. Data for optimal growth temperature were taken from the Engqvist dataset [58], BacDive, and Tempura [59], prioritized in this order. The temperature data were similarly matched with our dataset based on provided taxonomy ID, match to either strain, species, or genus ID in our dataset, whichever lowest was available. Bacterial species were classified into four categories, based on optimal growth temperature (if available): psychrophile (<20°C), mesophile (20°C–45°C), thermophile (45°C–60°C), and hyperthermophile (≥60°C). Gram-staining data were taken from BacDive.

### Protein sequence searches and gene neighborhood analysis

All polymerases were identified in proteomes using PSI-BLAST [60] (5 iterations, 1e-5 inclusion threshold) with multiple diverse queries for each DNA polymerase family. Each query sequence was trimmed to include only the conserved polymerase domain. Additional validation of the lowest-scoring results was done using HHpred [61] searches against PDB [62] and Pfam [63] databases. Sequences, in which the polymerase domain was not identified reliably (with over 90% probability), and fragments shorter than 200 residues, were discarded. Searches for additional proteins (PHP, DnaQ 3′–5′ exonucleases, Pol I-like 5′–3′ exonucleases, ImuB-C homologs) were performed in the same manner. Queries were trimmed to include only the domain of interest.

Gene neighborhoods were analyzed using GCsnap [64]. Protein grouping by sequence similarity was done using CLANS [65]. If needed, further validation of identified protein groups was performed with HHpred. The presence of integrated prophages in genomic sequences was analyzed using geNomad [66].

### Multiple sequence analysis

Initial multiple sequence alignments (MSAs) for sequence and motif analyses were constructed using fast Muscle5 algorithm [67]. Accurate final MSAs were derived with MAFFT [68], using the accuracy-oriented L-INS-i algorithm.

### Sequence classification and phylogenetic analysis

Sequence clustering, aimed to identify distinct groups, was done with CLANS. Phylogenetic tree construction was performed using IQ-TREE [69] (automatic model detection, 1000 replicates for construction and bootstrap). Strongly diverged or truncated sequences were discarded prior to the phylogenetic analysis. Prior to tree construction, MSAs were trimmed to include only selected domains (only polymerase domain for the PolA tree, PHP + polymerase domain for all C-family trees, only PHP domain for the PHP tree). Alignment columns with little information were removed using trimAl [70], leaving only columns with at least 70%–90% of sequences having an aligned residue in the column. The exact percentage in each case was chosen depending on which alignment produced a more reliable phylogenetic tree. Trees were displayed and annotated using iTOL [71].

### Identification of conserved motifs

Conserved sequence motifs for each protein group (cluster) were identified using profile–profile comparisons. Initially, representatives containing a sequence motif of interest (active site or interaction motif) were identified in each sequence cluster based on a known structure and/or literature. For each cluster, containing a representative sequence, an MSA was constructed, and only sequences that aligned over the motif were retained. Next, the MSA regions that included only the considered motifs (for very short motifs, adjacent regions were also included to increase the sensitivity of subsequent searches) were excised, and corresponding HMM profiles were generated using HMMER tools [72]. These HMM profiles were then queried against a database containing all full-length sequences analyzed in this work using hmmsearch. The obtained matches were additionally validated using reciprocal searches with HHsearch [73]. The identified sequence motifs were used to construct group-specific MSAs that, in turn, were converted into motif logos with WebLogo 3 [74].

### Protein structure modeling and analysis

Models of protein complexes without DNA were constructed using locally installed AlphaFold-Multimer [75] with default parameters and using default databases for MSA construction. For each complex, five models were produced, and the top-ranked one was selected. Models with DNA were constructed using a locally installed version of AlphaFold 3 [76]. Each top-ranked model was selected from 500 models (100 different seeds, 5 models per seed). Model reliability was assessed taking into account both local (pLDDT) and global (pTM and ipTM) AlphaFold confidence scores. Models were also assessed using VoroMQA [77] scores that are based on statistical potentials and therefore are complementary to AlphaFold scores. AlphaFold clash scores were checked to verify that models had no clashes. Finally, models were subjected to visual inspection to make sure that there are no remaining structural anomalies (e.g. knots). Interaction surface areas were calculated using VoroContacts [78]. Alignment and comparison of multiple structures were performed using Dali [79]. UCSF Chimera [80] was used for structure visualization and analysis.

### Statistical analysis

Metadata used for analyses included optimal growth temperature and oxygen usage. Growth temperature annotations were collapsed into two categories: thermophiles (including hyperthermophiles) and mesophiles (including psychrophiles). Oxygen usage was classified as aerobic or anaerobic. Organisms lacking annotation for the analyzed environmental variable (e.g. optimal growth temperature) were excluded from that analysis. The presence of a polymerase from the considered family/subset was encoded using a binary scheme (1 = presence, 0 = absence). Pairwise differences between environmental groups were assessed using two-sided Fisher’s exact tests applied to 2 × 2 contingency tables, yielding odds ratios with 95% confidence intervals and two-sided *P*-values. All statistical analyses were performed using SciPy and statsmodels Python libraries [81, 82].

## Results

**Figure 1.**
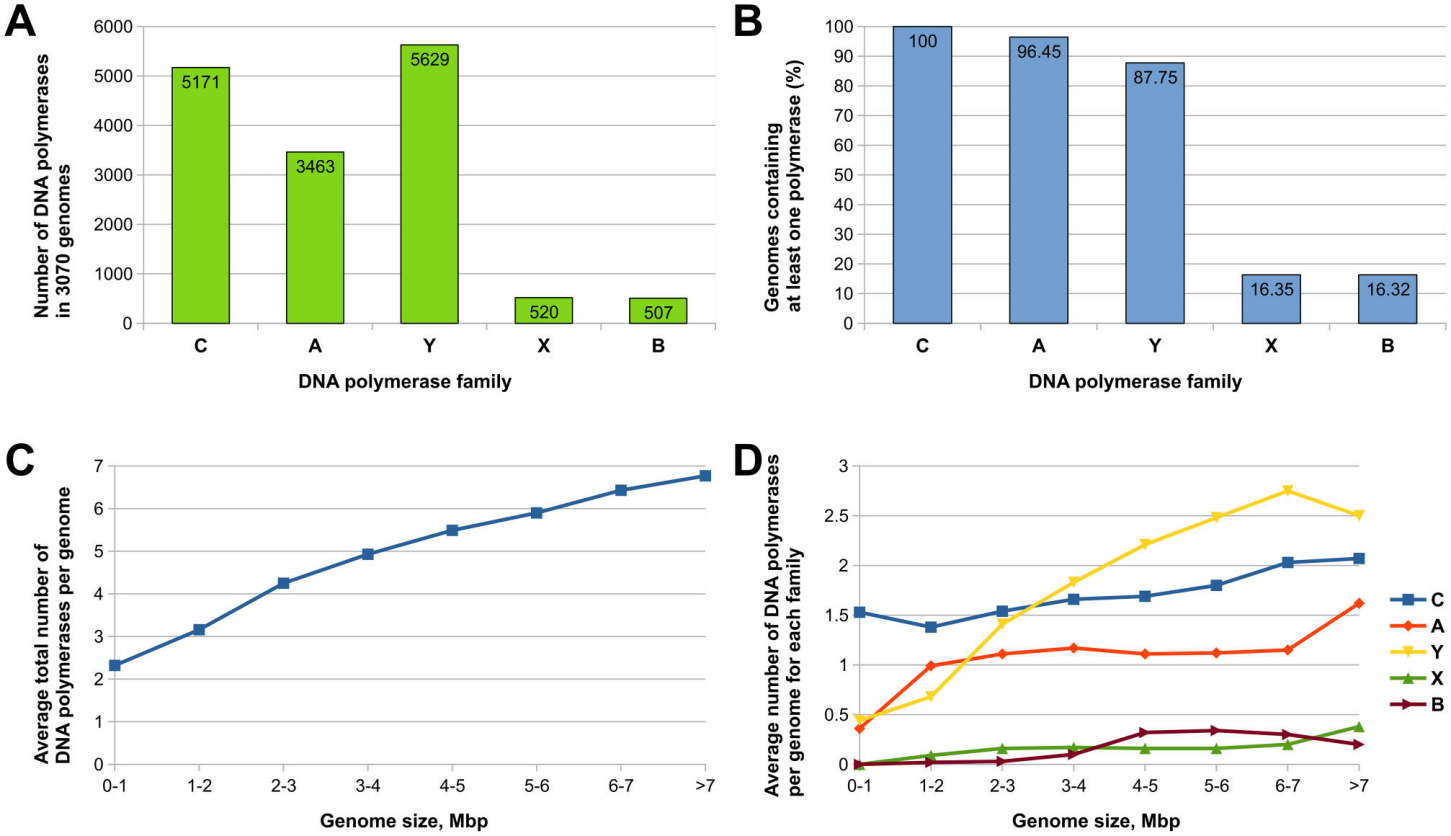
Abundance of DNA polymerases by family and genome size. (**A**) Total number of DNA polymerases of a given family in all genomes. (**B**) Percentage of genomes encoding polymerases of a given family. (**C**) Dependence of the total number of DNA polymerases on genome size. (**D**) Dependence of the number of polymerases for each family on genome size.

We analyzed 3070 complete “reference” and “representative” bacterial genomes and corresponding proteomes obtained from the NCBI [55] (Supplementary data file 1). This set represents a compact, normalized, and taxonomically diverse sample of available bacterial genome assemblies. First, we searched bacterial proteomes for DNA polymerases of A, B, C, X, and Y families. We found that Y-family, consisting of polymerases and catalytically inactive homologs, is the most abundant, followed by C and A families (Fig. 1A). However, the picture changes if we look at individual proteomes (Fig. 1B). C-family members are identified in every bacterial species, which is unsurprising given that the essential replicative DNA polymerases belong to the C-family. Polymerases of A-family are also present nearly universally (found in ~96% of species), whereas members of the most abundant Y-family are present in a somewhat lower fraction (∼88%) of species. The presence of B and X families in bacteria is not typical, as the members of either family were identified in only ~16% of species. Thus, although there are species (55 in our set) that have DNA polymerases from all five families, the most typical polymerase set in bacteria is composed of C-, A-, and Y-family members. Notably, in some taxonomic groups, DNA polymerase sets significantly deviate from this typical pattern (Supplementary Fig. S1).

We noticed that the overall number of DNA polymerases present in individual bacterial species directly correlates with the genome size, that is, the larger the genome, the more DNA polymerases it is likely to encode (Fig. 1C). The greatest contribution to this trend is made by Y-family members, except for the largest genomes, where the A-family contribution is also significant (Fig. 1D).

### C-family DNA polymerases comprise four major groups

**Figure 2.**
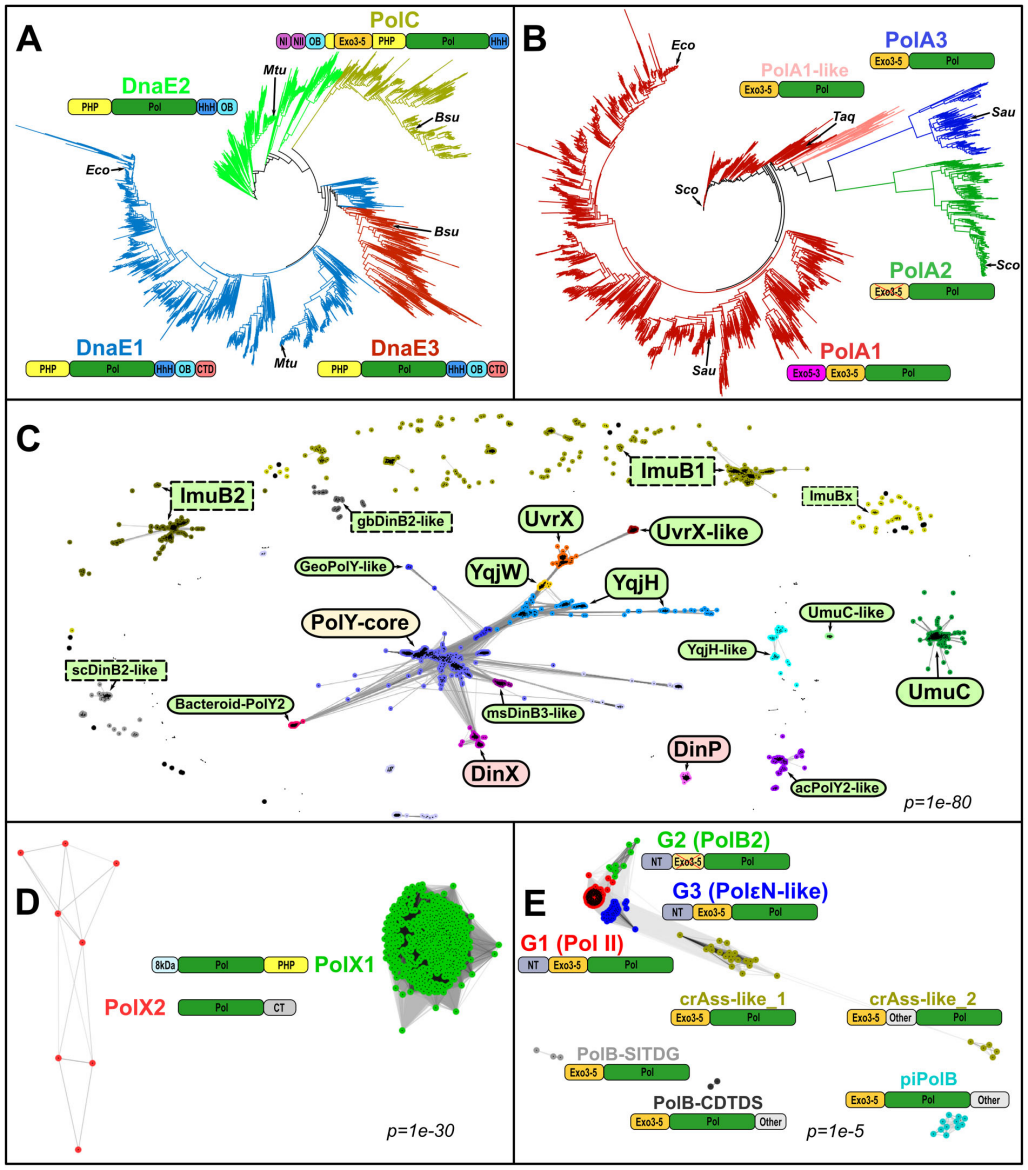
Diversity within individual DNA polymerase families. (**A, B**) Phylogenetic trees of C- and A-family, respectively. Major polymerase groups are represented by different colors and the most typical domain architecture. Domain labels: PHP, the polymerase and histidinol phosphatase domain; Pol, the polymerase core consisting of “palm,” “thumb,” and “fingers”; HhH, the tandem helix-hairpin-helix motif; OB, oligonucleotide binding domain; CTD, C-terminal domain; NI and NII, two N-terminal subdomains related to domain V of PolIII τ-subunit [83]; Exo3-5, DnaQ-like 3′–5′ exonuclease domain; Exo5-3, FEN-like 5′–3′ exonuclease domain. Species labels: *Eco, E. coli, Mtu, M. tuberculosis, Bsu, B. subtilis, Taq, Thermus aquaticus, Sco, Streptomyces coelicolor, Sau, Streptococcus aureu*s. The raw phylogenetic trees are available in Supplementary data file 3. (**C**–**E**) Sequence grouping of Y-, X-, and B-family, respectively, derived by CLANS clustering. For Y-family (C), the fill color of the labels indicates the RecA-NT motif prevalence in a given group: light green, most sequences have the motif; pink, no motif; yellow, partial motif presence. Groups dominated by active polymerases (catalytic aspartates identified) have labels with solid, rounded borders; inactive polymerases have labels with dashed rectangular borders. X-family (D) and B-family (E) groups are represented by their typical domain architecture. Domain labels: 8kDa, N-terminal lyase domain; CT, a novel C-terminal domain; NT, N-terminal domain; other, various other domains. Crossed 3′–5′ exonuclease domains in panels (B) and (E) denote catalytically inactive domains in PolA2 and G2 (PolB2) polymerases, respectively.

Consistent with previous studies [6, 7], phylogenetic analysis of C-family polymerases revealed four major groups: PolC, DnaE1, DnaE2 and DnaE3 (Fig. 2A and Supplementary data file 2). PolC and DnaE2 polymerase groups are resolved well, whereas the distinction between the DnaE-type Pol III α-subunits, DnaE1 and DnaE3, is not so clear-cut.

**Figure 3.**
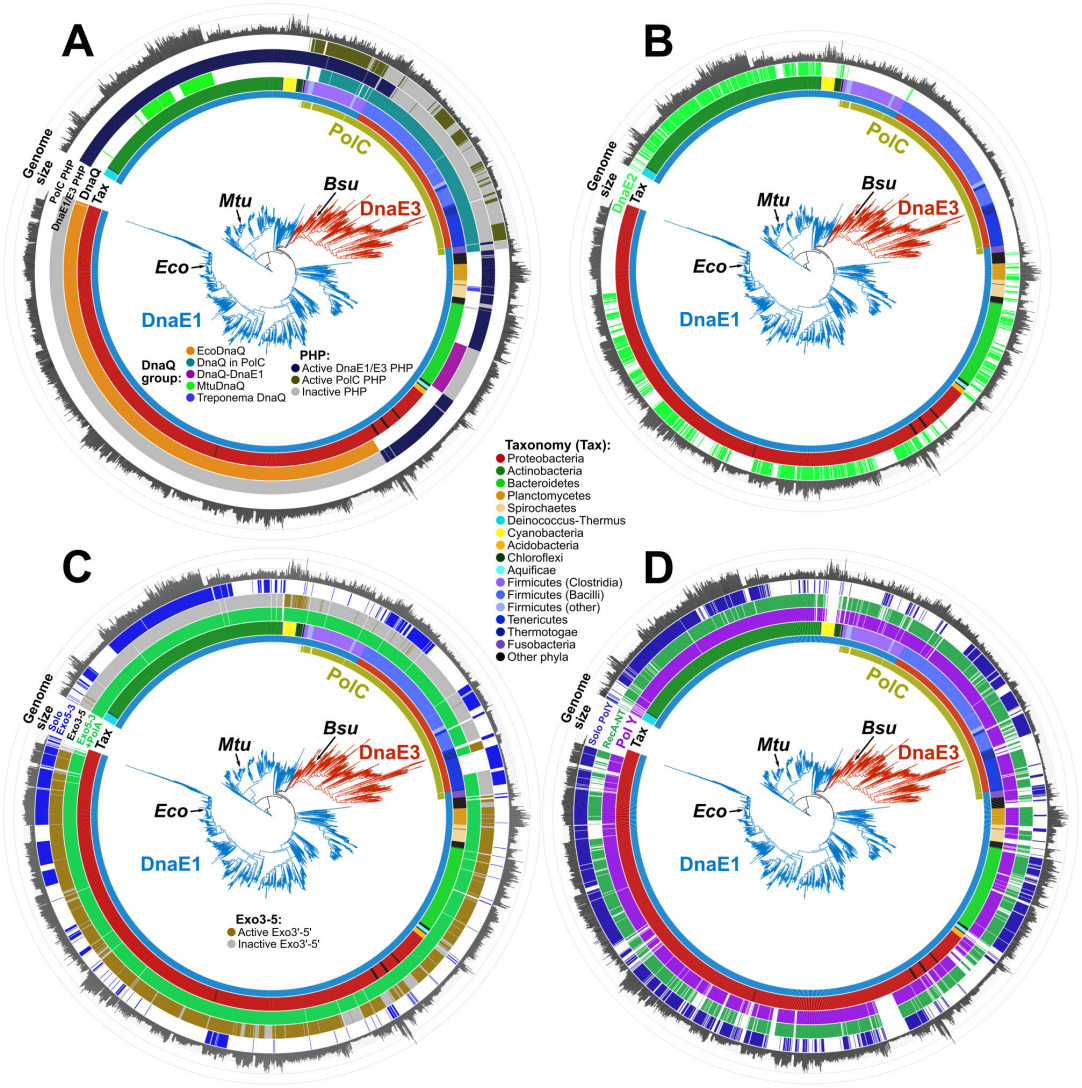
Mapping of various features on the bacterial species tree based on the DnaE1/E3 phylogeny. (**A**) Proofreading nucleases associated with replicative DNA polymerases of C-family: PHP and DnaQ domains of PolC and DnaE1/DnaE3 polymerases, and separate DnaQ subunits. (**B**) Accessory DnaE2 polymerases. (**C**) Nucleases associated with A-family: active FEN-like 5′–3′ exonuclease (Exo5-3) domains present as part of PolA1 (Pol I orthologs), active/inactive 3′–5′ exonuclease (Exo3-5) domains of PolA1, and standalone FEN-like 5′–3′ exonuclease (solo Exo5-3). (**D**) Presence of any Y-family members (PolY), only those with the RecA-NT motif (RecA-NT), and those without the motif (Solo PolY). The raw DnaE phylogenetic tree is available in Supplementary data file 3.

Previously, we showed that bacteria have one of the following three combinations of Pol III α-subunits: (i) DnaE1-only, (ii) PolC + DnaE1, and (iii) PolC + DnaE3 [7]. Taking advantage of the fact that every bacterial species has at least one Pol III catalytic subunit of the DnaE type (DnaE1 or DnaE3), we built a DnaE-based bacterial phylogenetic tree, which could be used to map various species-specific features (Fig. 3). As the grouping of DnaEs, co-occurring with PolC, into DnaE1 and DnaE3 may not be visually obvious from branch lengths alone, we explored it in more detail. In the tree, partitioning into DnaE1 and DnaE3 coincides with the split between two sharply contrasting classes of Firmicutes: Clostridia (anaerobic, PolC + DnaE1) and Bacilli (mostly aerobic, PolC + DnaE3). We built a tanglegram, in which the PolC tree was matched with the DnaE1/DnaE3 tree from the corresponding species, and found no edge crossings between the PolC + DnaE1 and PolC + DnaE3 systems, pointing to distinct evolutionary histories of these systems (Supplementary Fig. S2). Moreover, the signatures of the PHP active site and the clamp-binding motif sharply differ between clostridial DnaE1 and bacilli DnaE3 (Supplementary Fig. S3). Collectively, these observations support the DnaE1/DnaE3 split inferred in the tree.

Replicative C-family DNA polymerases, in addition to DNA synthesis, have a proofreading function associated with PHP domains and/or DnaQ domains/subunits. To investigate how these proofreading capacities are distributed, we assigned PHP domains as being “active” if they had the conserved active site motif (“HHDHEH[C/H]DH”) [7, 15, 84] or “inactive” if there were any deviation from this motif. We also identified DnaQ domains (embedded in PolC or fused to some DnaE1s) and separately encoded DnaQ subunits (Supplementary Fig. S4). DnaQ domains/subunits were considered “active” if they had the conserved “DEDDh” active site signature. Since we identified a large number of standalone DnaQ homologs (>3 per proteome on average), they were assigned as DnaQs only if they (i) were close homologs of the experimentally validated DnaQ proteins or (ii) produced high-confidence AlphaFold models for DnaE–DnaQ complexes. Using this conservative approach, we found that α-, β-, and γ-proteobacteria encode DnaQ proteins that are closely related to the *E. coli* Pol III ε-subunit, and most produce DnaE1–DnaQ models closely similar to the *E. coli* Pol III α–ε complex [85]. A group of proteins from Actinobacteria was assigned as DnaQ subunits based solely on their close homology to the recently characterized noncanonical mycobacterial DnaQ, acting synergistically with the PHP proofreader [86]. Consistent with experiments showing that mycobacterial DnaQ does not stably bind DnaE1 [15, 86], most AlphaFold models of mycobacterial DnaQ–DnaE1 complexes, in particular interaction interfaces, were of low confidence (typically, ipTM < 0.4). Finally, a small group of homologs from *Treponema* species were putatively assigned as DnaQ subunits. This assignment was based on the observation that models of corresponding DnaE1–DnaQ complexes had even higher confidence scores (pTM > 0.72, ipTM > 0.85) than *E. coli*-like DnaE1–DnaQ complexes. Complementary assessment of both *E. coli*-like and *Treponema* DnaE1–DnaQ models using VoroMQA produced global scores over 0.4, typical for high-accuracy experimental structures [77, 87] (Supplementary data file 4).

Mapping the identified proofreaders onto the DnaE tree (Fig. 3A) revealed that most bacteria with the DnaE1-only replicative systems have a single candidate proofreader represented by either PHP or DnaQ. Only close homologs of mycobacterial DnaQ and newly assigned putative *Treponema* DnaQ represent additional proofreaders. Strikingly, Clostridia (PolC + DnaE1 system) in most cases have three “active” proofreaders (PHP in PolC and DnaE1, and DnaQ in PolC). In the case of PolC + DnaE3 systems, proofreading is typically associated with DnaQ domain of PolC, while PHP domains of PolC and DnaE3 are often “inactive.”

We next looked at the distribution of non-essential error-prone DnaE2 polymerases. Consistent with their low fidelity, DnaE2 polymerases have non-conserved substitutions in the PHP active site (with only a few exceptions) and are not known to cooperate with DnaQ. In agreement with previous observations [7], DnaE2 almost exclusively occurs in species that encode DnaE1 as the only catalytic subunit of Pol III and that typically have fairly large genomes (Fig. 3B). DnaE2 polymerases are particularly common in Actinobacteria.

### DNA polymerases of A-family include Pol I orthologs and two other major groups

Both phylogenetic analysis and sequence clustering of A-family DNA polymerases revealed three major groups, which we denoted PolA1, PolA2, PolA3, and a minor group, PolA1-like (Fig. 2B, Supplementary Fig. S5, and Supplementary data file 2). The largest and the most widely dispersed group, PolA1, corresponds to the orthologs of *E. coli* Pol I, whereas PolA2 and PolA3 groups are significantly smaller and more compact. The nearly universal presence of A-family polymerases (detected in ~96% of genomes) is specifically due to Pol I orthologs.

Most PolA1 group polymerases have the canonical Pol I domain architecture, consisting of FEN-like 5′–3′ exonuclease, DnaQ-like 3′–5′ proofreading exonuclease and polymerase domains (Fig. 2B). The presence of the 5′–3′ exonuclease domain distinguishes PolA1 from other PolA groups that all lack this domain.

Previously, it has been shown that some bacteria can survive without the polymerase function of Pol I, but only if the 5′–3′ exonuclease activity is provided by either Pol I domain or a standalone protein [88]. We reasoned that in cases when canonical Pol I is absent or replaced by PolA1-like, the 5′–3′ exonuclease function might be supplied by a standalone (solo) nuclease. To test this idea, we searched bacterial proteomes for standalone homologs of the Pol I 5′–3′ exonuclease domain, and found them in most species lacking Pol I (Fig. 3C). Addition of these solo nucleases increased the overall coverage of the 5′–3′ exonuclease domain to 99.8% of the analyzed genomes (Supplementary Table S1 and Supplementary Fig. S6). Only five endosymbionts with tiny AT-rich genomes had neither Pol I nor a standalone 5′–3′ exonuclease domain. Taken together, these data indicate that the 5′–3′ exonuclease function might be dispensable only in bacterial species with extremely reduced genomes.

Interestingly, despite the nearly universal presence of PolA1 polymerases, about half of them have substitutions in the “DEDDy” active site motif of the 3′–5′ exonuclease, implying loss of the proofreading activity (Supplementary Fig. S7). Initially, we presumed that the catalytic inactivation of 3′–5′ exonuclease domains might be more common in bacteria with small genomes, but it turned out to be unrelated to genome size. Instead, the inactivation appeared to be largely phylum-specific, most common in Firmicutes, Tenericutes and Actinobacteria. (Fig. 3C and Supplementary Fig. S8). These observations suggest that, in contrast to the nearly universally conserved 5′-3′ exonuclease function, the proofreading function of Pol I might often be dispensable.

PolA2 polymerases are present only in Actinobacteria. This group was recently identified in a large-scale analysis and was named APEX (Actinobacterial Polymerases with a potentially Eclipsed eXonuclease) because of the inactivated 3′–5′ exonuclease domain [31]. Using sequence analysis, we discovered that PolA2 polymerases have a β-clamp binding motif (QxxLF) in the unstructured loop of the vestigial 3′–5′ exonuclease domain (Supplementary Fig. S9). To substantiate this putative PolA2-clamp interaction, we used AlphaFold to model a PolA2 complex with the β-clamp for *S. coelicolor* and several other species. The obtained high-confidence models revealed that the PolA2 clamp binding motif (120-QSSLF-124 in *S. coelicolor* PolA2, NCBI ID: CAD55317) is indeed bound to the conserved pocket of the β-clamp (Supplementary Fig. S9 and Supplementary data file 4). To our knowledge, PolA2 is the first bacterial A-family polymerase with the identified β-clamp binding motif. Thus, both previous [31] and our current findings suggest that PolA2 polymerases are low-fidelity polymerases capable of utilizing a DNA sliding clamp.

PolA3 polymerases, unlike PolA2, have an intact active site of the 3′–5′ exonuclease domain, suggesting that they are high-fidelity DNA polymerases (Supplementary Fig. S7). Also, unlike PolA2, PolA3 polymerases are scattered across diverse bacterial lineages, have closely related phage homologs (Supplementary Fig. S10), and are encoded in prophage regions suggesting viral origin.

### Y-family comprises highly diverse error-prone DNA polymerases and accessory proteins

Initially, we clustered Y-family members into distinct groups (Supplementary data file 2). The resulting grouping closely agreed with the one obtained previously [53], indicating the stability of the results (Fig. 2C and Supplementary Fig. S11).

Most of the Y-family groups comprise proteins with an intact polymerase active site, suggesting that they are active DNA polymerases. Proteins that lack catalytic residues are either dispersed or form diffuse groups. All of the PolY groups, with the exception of DinP, have β-clamp binding motifs displaying varying levels of similarity to the consensus “QxxLF” motif (Supplementary Fig. S11 and Supplementary data file 2). In addition, the absolute majority of both catalytically active and inactive PolY groups feature the RecA-NT motif, indicating that the corresponding proteins should be able to bind RecA or its homologs. Only four relatively small groups, including DinP, DinX, Fusobacteria-PolIV-like, and Betaprot-PolIV-like, completely lack the RecA-NT motif. Some of these groups (e.g. DinX) have additional domains or C-terminal tails that could potentially be involved in complex formation with other proteins. Members of the central group (PolY-core), typically regarded as orthologs of *E. coli* DinB, show a mixed pattern with the majority lacking the RecA-NT motif.

Next, we analyzed how common Y-family members with the RecA-NT motif (representing putative multimeric complexes) are among individual species. As can be seen in Fig. 1B, nearly 9 out of 10 bacterial genomes encode one or more members of Y-family. Bacteria that lack Y-family members typically have very small genomes, with Cyanobacteria representing a notable exception (Fig. 3D). Among the genomes encoding Y-family members, nearly 90% have at least one Y-family representative with the RecA-NT motif, whereas only 63% have representative(s) without this motif (solo PolY) (Fig. 3D). The distribution of the RecA-NT motif in Y-family is fairly uniform across the phylogenetic tree suggesting that this motif has already been present in an ancestral bacterial PolY polymerase. An alternative scenario whereby the RecA-NT motif was acquired multiple times independently by different bacterial phyla appears highly unlikely. Furthermore, the ubiquitous presence of the RecA-NT motif suggests the widespread involvement of RecA or its homologs in regulation of Y-family members.

### X-family DNA polymerases are rare and display low variability

Sequence clustering of X-family DNA polymerases revealed only two distinct groups, a major group (PolX1) and a minor one (PolX2), differing in domain architecture (Fig. 2D). The PolX1 group represents typical bacterial PolX proteins that have the 8 kDa N-terminal, polymerase, and PHP exonuclease domains, whereas PolX2 represents a novel group that lacks the N-terminal and PHP domains but has an additional α-helical C-terminal domain.

**Figure 4.**
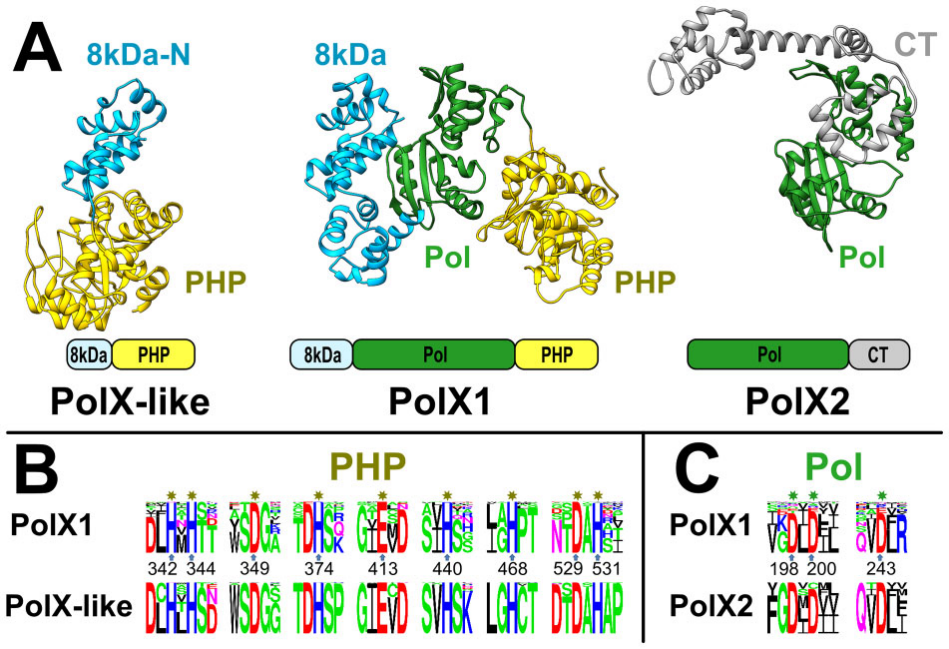
Comparison of PolX-like, PolX1, and PolX2. (**A**) *M. tuberculosis* PolX-like (model, AFDB ID: AF-P96221-F1), *T. thermophilus* PolX1 (PDB ID: 3AU2), and *Streptomyces hawaiiensis* PolX2 (model, AFDB ID: AF-A0A6G5RSZ6-F1) structures. Conservation of (**B**) PHP active site and (**C**) polymerase active site motifs. Residue numbering corresponds to *T. thermophilus* PolX1.

Upon examination of PolX1 sequences, we noticed that the PHP active site residues are visibly more conserved (95% of PolX1) compared to those in the polymerase active site (75% of PolX1), and that PolX1s with substitutions in the polymerase active site tend to separate from those that have both active sites conserved (Fig. 4 and Supplementary Fig. S12). Our observation is in line with the recent finding that a subset of prokaryotic PolX proteins have an inactivated polymerase active site but retain the 3′–5′ exonuclease activity of the PHP domain [46]. Collectively, these data suggest that, in the course of bacterial PolX1 evolution, there was a stronger selective pressure to preserve the PHP exonuclease function rather than to maintain the ability to synthesize DNA.

Prompted by the observed greater importance of the PHP active site compared to the polymerase active site in the PolX1 group, we decided to check whether there might be closely related PHP domains that are not part of PolX1. Using sequence searches, we indeed identified a group of proteins, which we named PolX-like, occurring almost exclusively in Actinobacteria. PolX-like proteins represent close PolX1 homologs that are specifically lacking part of the N-terminal 8kDa domain and the entire polymerase domain (Fig. 4 and Supplementary Fig. S13). This finding further signifies functional and/or evolutionary importance of the PHP domain in PolX1 and its homologs.

In contrast, PolX2 is missing the PHP domain altogether, while the polymerase active site is conserved (Fig. 4). To gain insight into the provenance of PolX2, we collected X-family homologs from all domains of life and viruses. Clustering of these homologs by sequence similarity revealed that PolX2 members are present not only in bacteria, but also in archaea, eukaryotes and viruses (Supplementary Fig. S14). Clustering results and the similarity of polymerase active site motifs are consistent with the scenario whereby PolX2 has evolved from bacterial PolX1 and spread horizontally to other cellular domains. The absence of the PHP domain suggests that PolX2 members comprise error-prone polymerases, yet their role remains enigmatic as none of PolX2 homologs have been studied experimentally.

### B-family polymerases are infrequent but diverse

Despite their scarcity in bacteria, B-family polymerases are surprisingly diverse. Grouping by sequence similarity produced eight distinct groups, which we mapped to the previously defined B-family groups [32] by clustering all of them together (Fig. 2E, Supplementary Fig. S15, and Supplementary data file 2). We also identified a typical domain architecture of each group (Fig. 2E and Supplementary Fig. S16). The first three groups (G1–G3) have the canonical architecture, including the N-terminal, DnaQ-like 3′–5′ exonuclease and polymerase domains [34]. The G1 group, representing *E. coli* Pol II and its orthologs, is by far the largest one comprising ~80% of all B-family polymerases in our set of bacterial proteomes. The second most abundant group (G3) includes experimentally uncharacterized polymerases, related to a group of archaeal polymerases and to the N-terminal region of eukaryotic Polε [32]. Both of these PolB groups have conserved 3′–5′ exonuclease and polymerase active sites as well as a β-clamp binding motif, the features that are characteristic of high-accuracy DNA polymerases capable of processive DNA synthesis. In contrast, polymerases of the G2 group, recently proposed to form a novel mutasome complex [40], have an inactivated DnaQ-like domain. Remaining minor groups, also identified previously [32], all lack the N-terminal domain, most have intact DnaQ-like proofreader and some have additional domains.

### DnaE2 and Y-family members participate in diverse mutasome complexes

Our earlier study of Y-family suggested that, in addition to known Pol V and ImuA–ImuB–DnaE2 mutagenic complexes, there may be various other multimeric TLS polymerases [53]. Here, we systematically analyzed conserved associations involving DnaE2, Y-family members possessing the RecA-NT motif or both. Initially, we used conserved co-localization and co-occurrence patterns to identify known and to predict novel associations. We then used AlphaFold modeling to test whether these identified associations may correspond to multimeric complexes.

#### DnaE2-based association modules extend beyond canonical ImuA–ImuB–DnaE2 systems

**Figure 5.**
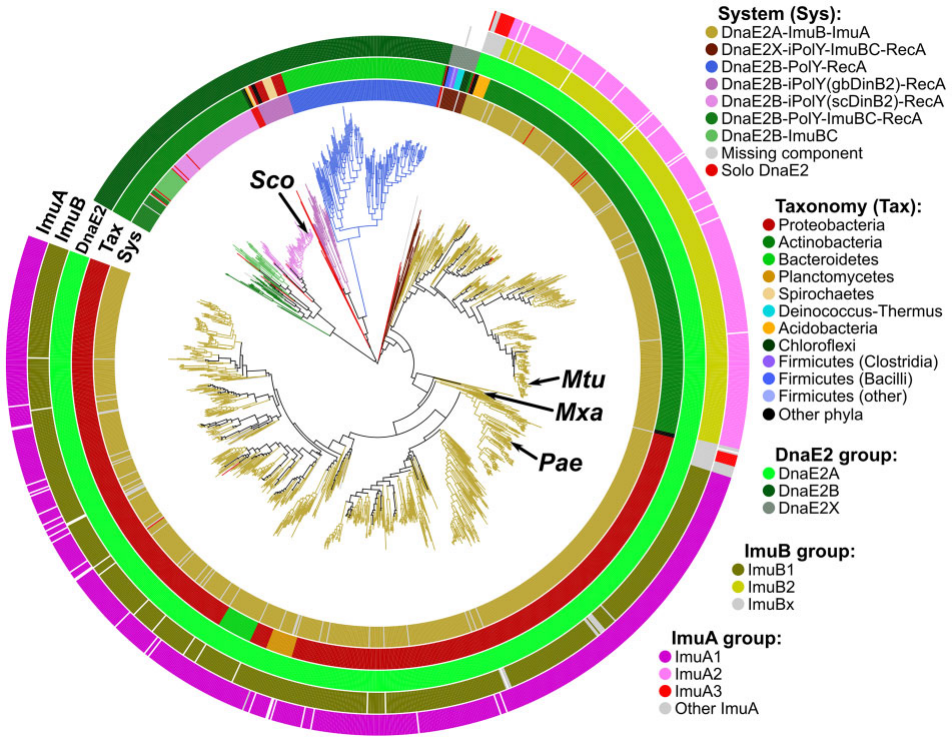
Phylogenetic tree of DnaE2 polymerases. The tree and the inner ring (Sys) are colored by the known or predicted composition of multimeric DnaE2 systems. The “Tax” ring is colored by taxonomy. Coloring of the “DnaE2,” “ImuB,” and “ImuA” rings reflect distinct groups within the corresponding protein family. Labeled representatives*: Sco, S. coelicolor, Mtu, M. tuberculosis, Mxa, M. xanthus, Pae, P. aeruginosa*. The raw DnaE2 phylogenetic tree is available in Supplementary data file 3.

We found that DnaE2 associations with other proteins largely correlate with the DnaE2 phylogeny. We classified DnaE2 into three groups, DnaE2A, DnaE2B, and DnaE2X. DnaE2A is the largest group and the only one we found associated with ImuA and ImuB as part of the ImuA–ImuB–DnaE2 module (Fig. 5). Notably, ImuA and ImuB families are not homogeneous either, and each can be subdivided into at least three groups with their boundaries closely matching on the DnaE2 tree. The two largest co-occurring ImuA and ImuB groups (ImuA1–ImuB1 and ImuA2–ImuB2) are predominantly found in two large taxonomic groups, Proteobacteria and Actinobacteria, respectively. ImuA1 and ImuA2 groups lack Walker A and B motifs that define the ATPase active site in RecA and, therefore, are expected to be catalytically dead. In contrast, ImuA3 possesses both motifs, suggesting it may comprise active ATPases closely related to RecA (Supplementary Fig. S17). Indeed, a recent study showed that ImuA from *Myxococcus xanthus*, which we assigned to the ImuA3 group, is an ATPase enhanced by DNA [89]. ImuB1 and ImuB2 groups can be easily distinguished, while the remaining ImuBs (ImuBx) show similarity to both, but most are closer to ImuB1 (Supplementary Fig. S18). In addition to overall sequence differences, distinct ImuB groups feature differences in the RecA-NT and β-clamp binding motifs, which are accompanied by differences in the DnaE2 motifs. The observed co-evolution of DnaE2, ImuA, and ImuB further supports the notion that all three proteins function together as a multimeric complex.

Unlike DnaE2A, members of another major DnaE2 group (DnaE2B) are found in genomes that do not encode ImuA or ImuB. Moreover, compared to DnaE2A, DnaE2B polymerases show significantly more diverse associations. They are often associated with active or inactivated PolYs that have RecA-NT motif, suggesting that RecA is also part of the putative multimeric complex (Fig. 5). One of the larger DnaE2B groups, found in Bacteroidetes, is linked to an active Y-family polymerase and RecA (DnaE2B-PolY-RecA). Another group, identified in Actinobacteria, corresponds to DnaE2B coupled with an inactivated Y-family polymerase (iPolY) and RecA (DnaE2B-iPolY-RecA). This system is mostly confined to *Streptomyces*, where iPolY corresponds to DinB2 (scDinB2), often present in the same operon with DnaE2 [90]. The third DnaE2B group is represented by a putative system of identical composition (DnaE2B-iPolY-RecA), but it has a wider phyletic spread and iPolY belongs to a different group of iPolYs, represented by *G. bemidjiensis* DinB2 (gbDinB2) [53, 90]. The fourth DnaE2B group is associated with apparently active PolY, a standalone protein of the ImuB-C family, and RecA. One of the representatives of this system is present in *Tessaracoccus lapidicaptus*. The fifth DnaE2B group, exemplified by *Arthrobacter alpinus*, is linked only to ImuB-C. For a few DnaE2Bs, we were unable to find any associated partners.

The third DnaE2 group, a small but taxonomically diverse DnaE2X group, is associated with iPolY, ImuB-C, and RecA. As iPolY and ImuB-C may be considered to represent a split ImuB, this type of multicomponent system most closely resembles the ImuA–ImuB–DnaE2 module. Taking these observations into account, the DnaE2X systems might be considered to represent an ancestral precursor for the more abundant DnaE2A systems.

#### DnaE2-independent PolY associations involve RecA and diverse accessory factors

We also explored conserved associations of Y-family groups that do not partner with DnaE2 but have the RecA-NT motif. Consistent with previous results [53], we identified homologs of *E. coli* Pol V, homologs of a putative *B. subtilis* complex consisting of YqjW (PolY2), YqjX (UmuD analog), and RecA, some PolYs associated with ImuB-C, and various putative PolY–RecA complexes. In addition, analysis of smaller Y-family groups revealed a specific link between UmuC-like polymerases and a group of proteins related to the previously identified SRAP (SOS response associated peptidase) superfamily [91].

#### AlphaFold modeling supports the view that DnaE2- and PolY-based association modules correspond to protein complexes

**Figure 6.**
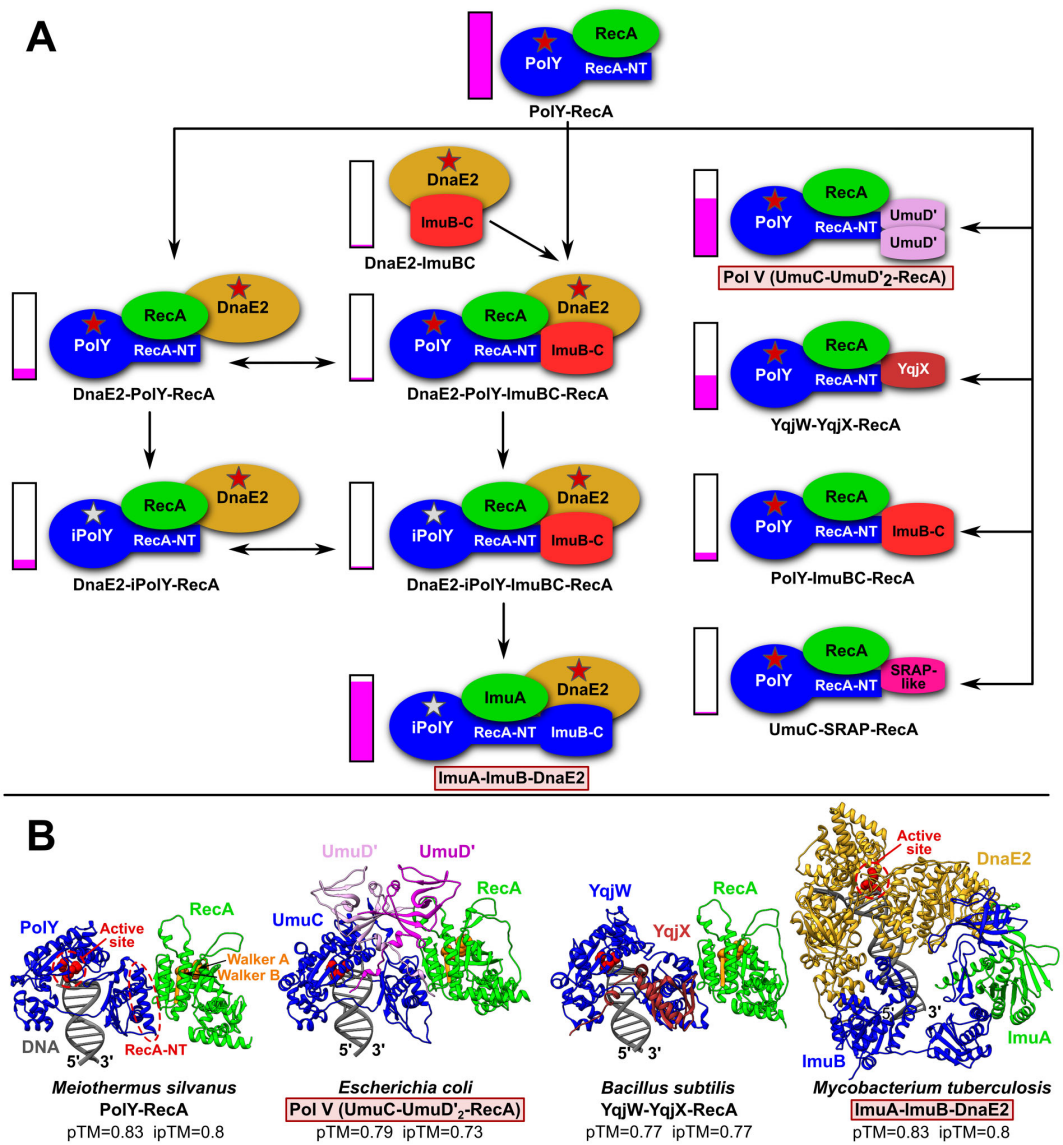
Known and predicted association modules corresponding to multisubunit complexes formed by DnaE2 (DnaE2A/B/X) and/or PolY DNA polymerases. Framed labels with a pink background indicate known complexes. (**A**) Composition of 11 types of complexes identified in this study. Different individual subunits are shown in different colors. For Y-family members, the polymerase region (PolY/iPolY), the RecA-NT motif, and the ImuB-C domain are labeled individually. Red and gray stars represent canonical and disrupted active sites, respectively. Arrows indicate possible evolutionary steps leading to various architectural solutions, starting from the presumed ancestral PolY–RecA complex. Columns with the magenta filling represent relative abundance of each system compared with the PolY-RecA dimer, which is the most abundant. (**B**) Structural models of the four most common types of multisubunit error-prone DNA polymerases.

Altogether we identified 11 types of DnaE2- and PolY-based association modules, only two of which (Pol V and ImuA–ImuB–DnaE2) have been characterized previously (Fig. 6A). Based on the recurring pattern of some module components, we proposed evolutionary steps that may have led to the emergence of the observed types of modules. We next asked whether all these association modules might represent protein–protein complexes. To this end, taking several representatives for each type, we generated corresponding structural models using both AlphaFold-Multimer (for protein-only complexes) and AlphaFold 3 (for protein complexes with DNA). Representatives of all 11 association types produced models with AlphaFold ipTM scores well above independently established thresholds (0.65 for AlphaFold-Multimer; 0.7 for AlphaFold 3) for complex models expected to be correct [92]. Furthermore, a number of models within different complex types had pTM and ipTM scores ≥ 0.8, denoting high-confidence predictions (Fig. 6B, Supplementary Fig. S19, Supplementary Table S2, and Supplementary data file 4). Assessment with VoroMQA produced favorable global scores (>0.4) for all models. In addition, structural models (both with and without bound DNA) for each type of module from different species showed high consistency in overall shape and interaction interfaces. Taken together, these data suggest that all the identified association modules correspond to protein complexes and that their structural models are fairly accurate.

The abundance of identified protein complexes in our set of bacterial species is highly unequal (Fig. 6A). Since this set is representative, it might reflect the relative overall abundance of these mutasome types in bacteria. Figure 6B shows selected AlphaFold structural models of the four most abundant types of multisubunit error-prone DNA polymerases that make up ~90% of all multimeric mutasomes.

The first of the four complexes, PolY-RecA, corresponds to the proposed ancestral multimeric PolY mutasome with the simplest architecture. This type of putative association is seen in a number of PolY subfamilies and many different taxonomic groups. RecA binding presumably may regulate the polymerase activity and/or mutational profile of PolY. Despite extremely wide distribution, so far, none of such complexes has been characterized experimentally.

The second complex, consisting of UmuC, a UmuD′ dimer, and RecA, is exemplified by the extensively studied *E. coli* Pol V mutasome. Recently, we published a Pol V structural model [40], providing a detailed picture of interactions within the complex. Briefly, UmuC, through its RecA-NT motif, interacts with RecA, whereas the very C-terminal region of UmuC binds one of the UmuD′ dimer subunits. Another UmuD′ subunit inserts its N-terminal arm into the cleft formed by Palm and Little Finger (LF) domains of UmuC, such that the very N-terminus of UmuD′ is positioned close to the UmuC active site.

The third mutasome type is exemplified by the putative YqjW-YqjX-RecA (and closely related UvrX-YolD-RecA) complex from *B. subtilis*. It has an architecture similar to Pol V, but details differ. YqjX/YolD, an UmuD analog, is likely to be present in the complex as a monomer, not as a dimer like UmuD′. We performed modeling experiments in which we attempted to produce models of dimeric and higher oligomeric states of YqjX, either alone or in the context of a full complex, but resulting models typically produced non-interacting YqjX monomers. This suggests that the YqjW–YqjX–RecA complex is a heterotrimer with each subunit present as a single copy. As expected, the YqjW–RecA interaction is very similar to the UmuC–RecA interaction. On the other hand, YqjW has a shorter C-terminal tail, which does not interact with YqjX. Similar to UmuD′, the N-terminal arm of YqjX is inserted into the YqjW, but its interaction interface with YqjW is about twice as large as in the case of UmuD′ N-terminal arm interaction with UmuC (Supplementary Table S3).

Surprisingly, based on gene neighborhood analysis, we found that there are at least three variants of this putative mutasome type (YqjW–YqjX–RecA) that differ by the nature of a small subunit (Supplementary Fig. S20). The most common subunit is a typical YqjX/YolD protein, the second one corresponds to just the N-terminal region of YqjX/YolD, and the third one, exemplified by *Cohnella abietis* YhjD, represents a homologous N-terminal region fused to a domain unrelated to YqjX. Strikingly, all three accessory subunits are linked only by the homology of the N-terminal arm, which in all cases is similarly inserted into the PolY polymerase of the YqjW/UvrX type.

The fourth complex is represented by a structural model of the previously established *M. tuberculosis* mutasome, minimally comprising ImuA, ImuB, and DnaE2 [19, 52]. The model revealed that the ImuB C-terminal region (ImuB-C), adjacent to the RecA-NT motif, represents a β-barrel. Interestingly, this β-barrel is not structurally similar to the SH3 β-barrel shared by UmuD′ and YqjX (Supplementary Fig. S21). Moreover, we were unable to find closely related structures in the PDB, suggesting that ImuB-C represents a novel structural fold. ImuB binds to ImuA through the RecA-NT motif similarly as *E. coli* UmuC binds to RecA, but the interaction is more extensive as the ImuB-C domain also contributes to ImuA binding. Nevertheless, a recent study has shown that the RecA-NT motif is critical for the ImuB–ImuA interaction and mutasome function [54]. DnaE2 interacts with ImuB primarily via two distinct regions. The most extensive interaction interface is between the PHP domain of DnaE2 and the C-terminal β-barrel domain of ImuB with some contribution from an extended ImuA loop. Another interface is formed between the DnaE2 region, annotated as “(HhH)_2_” or “β-binding domain” in DnaE1 [93, 94], and the “thumb” domain of ImuB. Proteins of both ImuB groups (ImuB1 and ImuB2) are quite variable and many of them feature deletions of various structural elements, in some cases resulting in drastically reduced structures (Supplementary Fig. S22). However, even the most reduced ImuB structure retains the ImuB-C domain and two other structural elements, the “Little Finger” (LF) and “thumb.” Notably, “thumb” and ImuB-C mediate interaction with DnaE2, and their evolutionary conservation provides further support for AlphaFold structural models of ImuA–ImuB–DnaE2 complexes.

### Taxonomy, replication systems, and environmental categories shape DNA polymerase distributions

**Figure 7.**
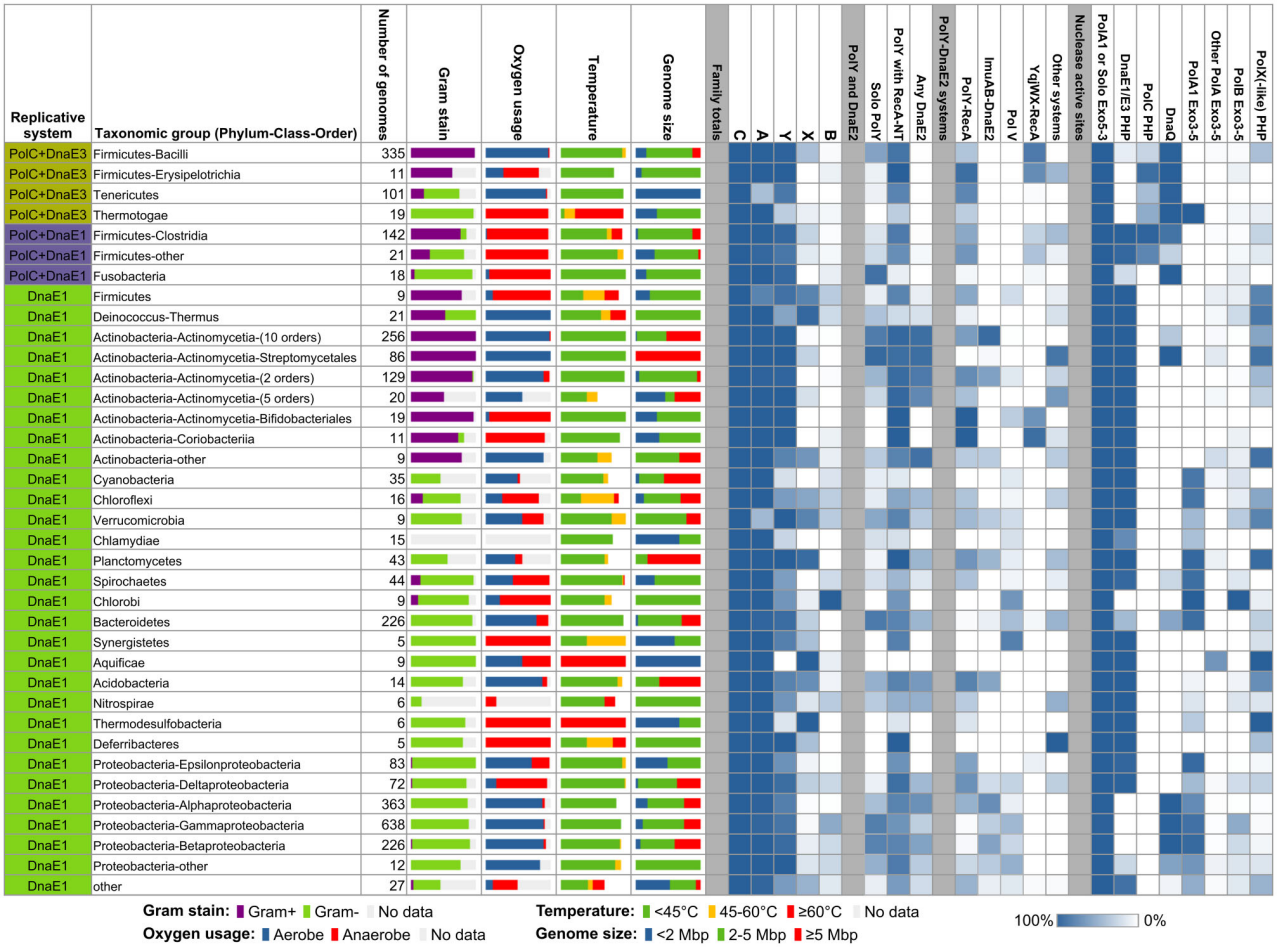
Summary of DNA polymerase groups, domains, and motifs identified in various taxonomic groups. The heat map indicates the percentage of members of a given taxonomic group that have at least one instance of the indicated feature. Nuclease active site motifs considered as “active”: Exo5′–3′, “D[D/E]EDD”; PHP, “HHDHEH[C/H]DH”; Exo3′–5′, “DEDDy.” “DnaQ” includes any of the DnaQ groups with the “DEDDh” active site shown in Fig. 3A. “PolX(-like) PHP” denotes active PHP domains of PolX and PolX-like proteins.

Based on detailed sequence and structure annotation of groups within each DNA polymerase family, we compiled a comprehensive account of polymerase distribution patterns. We have further linked these patterns with genome characteristics (size, GC%) and environmental factors (oxygen availability, temperature). Additionally, we included Gram staining annotations. The summarized results are presented in Fig. 7, with details provided in Supplementary data file 5.

One of the obvious observations is that the distribution of DNA polymerase families and their specific groups, multimeric polymerase complexes, and polymerase-associated nuclease domains are largely lineage-specific.

DNA polymerases of abundant A and Y families show noticeable depletion in some lineages. A significant fraction of species from Tenericutes and Verrucomicrobia lack Pol I orthologs (PolA1). However, in all such cases, they encode a homologous standalone FEN-like 5′–3′ exonuclease presumably fulfilling the role of the corresponding domain of missing Pol I. Y-family members are either absent or depleted in Thermotogae, Aquificae, and Thermodesulfobacteria, comprising bacteria typically living in high-temperature environments, as well as in mostly aerobic, mesophilic Cyanobacteria and Chlamydiae.

Although X and B families are poorly represented overall, some lineages show a visible enrichment in members of these families. Thus, all Deinococcus-Thermus, Aquificae, and Thermodesulfobacteria species and over 90% of Planctomycetes have X-family representatives. In addition, several other phyla show moderate X-family enrichment. B-family members appear to be mostly scattered among different phyla with only Chlorobi sticking out. In this phylum all species have a copy of PolB belonging to the uncharacterized G3 group.

Among abundantly represented phyla, Actinobacteria stand out for having multiple polymerases from the C, A, and Y families, while typically lacking B- and X-family polymerases (Supplementary Fig. S23). Some polymerase groups are found only in this phylum. For example, only Actinobacteria encode PolA2, featuring an inactive 3′–5′ proofreading domain and a β-clamp binding motif. Actinobacteria also possess unique groups of Y-family polymerases, including DinX/DinB1 with the C-terminal Tudor domain [53], DinP/DinB2, naturally adept at incorporating ribonucleotides [95], homologs of *Mycobacterium smegmatis* DinB3 (msDinB3-like), and a PolY group specific to Coriobacteriia. In addition, Actinobacteria have distinct ImuA and ImuB subunits (ImuA2 and ImuB2) of the ImuA–ImuB–DnaE2 mutasome and a distinct inactive PolY (scDinB2-like) in the putative iPolY–DnaE2–RecA complex. Other putative DnaE2-based complexes, DnaE2-[ImuB-C] and DnaE2-[ImuB-C]-PolY-RecA, are also found only in Actinobacteria. Likewise, PolX-like proteins lacking the polymerase region occur almost exclusively in Actinobacteria.

Next, we considered how the distribution of multimeric polymerase complexes, occurring in multiple bacterial lineages, relates to the three replication systems. Of the four major mutasome types, the most abundant PolY–RecA systems are present in species with any type of replication system. Putative mutasomes of the YqjW–YqjX–RecA type were also found co-occurring with all three replication systems, but in much fewer taxonomic groups. They are most abundant in Gram-positive bacteria with the PolC + DnaE3 replication system. In contrast, we identified Pol V and ImuA–ImuB–DnaE2 only in bacteria with DnaE1-only replication systems. Despite previous assertions that DnaE2 replaces Pol V [20, 96], we found a significant number of species having Pol V alongside ImuA–ImuB–DnaE2 and other DnaE2-based complexes. Interestingly, DnaE2-iPolY-ImuBC-RecA is the only DnaE2-based complex found in bacteria with all three replication systems. As its architecture is the most similar to ImuA–ImuB–DnaE2, this observation supports the idea that DnaE2-iPolY-ImuBC-RecA may represent an evolutionary predecessor of ImuA–ImuB–DnaE2 (Fig. 6A).

Finally, we explored associations between polymerase distributions and environmental variables (temperature and oxygen). We divided bacteria into thermophiles/mesophiles and aerobes/anaerobes and then checked for statistically significant differences in polymerase presence within each pair of groups using two-sided Fisher’s exact tests (Supplementary Fig. S24 and  Supplementary Table S4). Since A-family members (specifically PolA1) are present nearly universally, we focused only on two informative PolA1 subsets: with the active and the inactive 3′–5′ proofreading domain (Fig. 3C). For Y-family, we considered all polymerases, those with the RecA-NT motif, and solo PolY lacking the motif (Fig. 3D). In the case of sparsely represented X and B families, we considered any member of that family. In Y-family, solo PolYs are strongly enriched in mesophilic aerobic bacteria, whereas the same trend is weaker if we consider any PolY or those with the RecA-NT motif. X-family (dominated by PolX1) is enriched in thermophiles irrespective of their oxygen usage status. PolA1 polymerases with the inactive 3′–5′ exonuclease domain are enriched in anaerobes, whereas those with the active domain are enriched in aerobes. Both groups show a weaker association with the growth temperature. PolB polymerases show moderate enrichment in aerobes. Taken together, these results suggest that the environmental factors shape the distribution of at least some polymerase families or their groups.

## Discussion

There have been multiple studies of individual DNA polymerase families revealing structurally and functionally distinct groups within a given family [7, 31, 32, 46, 53]. However, the DNA synthesis capacity of a cell, required for DNA replication and maintenance, depends on the entire collection of DNA polymerases in the cell. Our study is the first attempt to identify patterns of DNA polymerase distribution within bacterial species and to explore whether these patterns correlate with global characteristics of the cells and/or their habitats.

Our results revealed that in bacteria, C, A, and Y polymerase families are the most common, whereas B and X are not, except for specific bacterial lineages. In general, the number of DNA polymerases in a given species strongly and positively correlates with the genome size. The highest contribution to this trend comes from Y-family DNA polymerases. This is not surprising as Y-family polymerases are naturally adept at TLS through DNA lesions caused by both endogenous and/or exogenous DNA-damaging agents [51].

Although the primary function of DNA polymerases is DNA synthesis, there are groups in all DNA polymerase families, except for the Y-family, endowed with additional 5′–3′ and/or 3′–5′ exonuclease activities. The 5′–3′ exonuclease is associated with the N-terminal FEN-like domain of PolA1 (Pol I orthologs), whereas the 3′–5′ proofreading exonuclease activity can be provided by either the PHP domain (C and X families) or the DnaQ-like exonuclease (A, B, and C families). The latter is found either as an intrinsic domain of a polymerase subunit or as in *E. coli*, may be encoded as a separate protein. Our results, based on the observed evolutionary conservation, revealed that in some cases these additional enzymatic activities appear to be more important than the polymerase activity itself.

The 5′–3′ endo/exonuclease activity, required for processing 5′-flaps during Okazaki fragment maturation, represents a case in point. This enzymatic activity is carried out by the FEN-like 5′–3′ exonuclease domain of Pol I. Previously, it has been shown that some bacteria could survive without the polymerase function of Pol I, but only if either the intact 5′–3′ exonuclease domain of Pol I was preserved or a standalone homolog was available [88]. We found that this might be a nearly universal requirement, because if a Pol I ortholog was missing, in all cases, except five, we found a complementing standalone FEN-like nuclease.

A clear case of the 3′–5′ exonuclease being more important than the polymerase is represented by the PHP domain of the main PolX group (PolX1). In agreement with a recent large-scale analysis of prokaryotic PolX polymerases [46], our results show that in PolXs the PHP 3′–5′ exonuclease active site is conserved stronger than the polymerase active site. In addition, we discovered PolX-like proteins that are highly similar to PolX1 but lack the polymerase region. Although the role of PolX-like proteins remains to be established, this observation provides further support to the idea that PolX and PolX-like proteins might play a more important role in processing DNA intermediates during DNA repair than in DNA synthesis [46]. Our results show that bacterial phyla associated with harsh living environments such as high temperature or desiccation, potentially promoting DSBs, are enriched in PolX proteins.

In remaining cases, the conservation of the 3′–5′ proofreading exonuclease active site in either PHP or DnaQ-like domains is generally lower than that of the polymerase. Different fractions of inactivated PHP proofreaders are present in all four major C-family groups (DnaE1–3 and PolC). It is not surprising that DnaE2 polymerases, with few exceptions, have inactivated PHP domains, as they represent non-essential SOS-inducible TLS polymerases [18, 19]. Perhaps it is more surprising that the PHP active site is also disrupted in most DnaE3, and in a fraction of DnaE1 and PolC polymerases, all representing essential Pol III α-subunits. However, there seem to be various solutions to compensate for the defective PHP proofreading function in replicative polymerases. In DnaE3, it can be complemented *in trans* by the DnaQ domain of PolC as observed in *B. subtilis* [97]. Inactive PHP domains in PolC and in DnaE1 are complemented by either the intrinsic DnaQ domain or a standalone DnaQ proofreader like in *E. coli* [17]. In Chlorobi and a few other bacteria with the inactive PHP domain of DnaE1, we failed to identify canonical standalone DnaQ exonucleases. However, given the importance of Pol III proofreading function, these bacteria may possibly use noncanonical proofreaders. Interestingly, in Pol III α-subunits it is the PHP domain that is inactivated and not the DnaQ proofreader. In contrast, the DnaQ-like domains with disrupted active sites are observed in a large fraction of Pol I orthologs. Such inactivation might be important for Pol I involvement in error-prone DNA repair as exemplified by the *Helicobacter pylori* and *B. subtilis* Pol I enzymes that participate in TLS [98, 99]. *Bacillus subtilis* Pol I was also found to be involved in TLS together with Y-family polymerases [99]. Whether such functional coupling between PolYs and Pol I orthologs with the inactivated 3′-5′ proofreader is widespread remains to be investigated. DnaQ-like domains are also inactivated in all G2/PolB2 polymerases and PolA2s. Consistent with this observation, PolB2 representatives in archaea were found to be error-prone and to function primarily as extenders in TLS [38, 39]. Although the cellular role of PolA2, also known as APEX [31], remains to be established, our observation that the appearance of PolA2 in Actinobacteria with large genomes coincides with a lower-than-expected number of PolYs strongly suggests the involvement of PolA2 in mutagenic DNA repair.

In agreement with previous observations [53], we found that the Y-family comprises multiple diverse groups of both catalytically active DNA polymerases and their homologs with disrupted active sites, both often featuring RecA-NT motifs. Here, our results showed that if a species has Y-family members, at least one of them is likely to have the RecA-NT motif. Furthermore, Y-family members with the RecA-NT motif are spread fairly evenly throughout bacterial lineages, suggesting that PolYs with the RecA-NT motif have already existed in ancestral bacterial species.

Besides findings related to individual polymerase families, our results significantly expanded the repertoire of putative multimeric error-prone DNA polymerases. In addition to well-known Pol V and ImuA–ImuB–DnaE2 mutasomes, we identified a number of novel putative multimeric complexes. Some of these complexes, similarly to Pol V, include PolY complexed with RecA and a distinct small subunit. We have previously proposed that in *B. subtilis* and other Gram-positive bacteria, the role of a small regulatory subunit is played by a YqjX/YolD family protein [53]. Here, we unexpectedly found that there may be other PolY-associated small subunits related to the YqjX/YolD family only by the common unstructured N-terminal arm, which interacts with PolY similarly to how the UmuD′ N-terminal arm interacts with UmuC in Pol V [40]. Other identified putative PolY-binding regulatory subunits include ImuB-C and SRAP-like proteins that were previously implicated in DNA damage response [91]. The analysis of DnaE2 also brought surprises. Earlier, it was assumed that DnaE2 is always part of the ImuA–ImuB–DnaE2 module, even prompting the proposal to rename DnaE2 to “ImuC” [20]. However, our results show that DnaE2B and DnaE2X, representing a significant fraction of DnaE2s, are encoded in genomes that lack *imuA* and *imuB* genes. Instead, these DnaE2s were found to be associated with various active or inactive PolYs, all capable of RecA binding. Some other DnaE2s showed association only with ImuB-C. In light of these observations, the use of “ImuC” to refer to DnaE2 seems to be misleading. A putative PolY-RecA dimer is the most abundant and most widely dispersed, suggesting that it represents an ancestral complex, which potentially gave rise to many other PolY-based error-prone DNA polymerase complexes. This also implies a prominent role of RecA in the regulation of Y-family polymerases.

Collectively, our comprehensive annotation of species-based DNA polymerase sets provides detailed information on the DNA synthesis and proofreading arsenal in bacteria. Our analysis showed that the polymerase complements are predominantly specific to individual bacterial lineages. However, distribution patterns of some multimeric polymerases transcend lineage boundaries and instead show dependence on DNA replication systems. Furthermore, our statistical analysis revealed that some polymerase families/groups are enriched in different temperature or oxygen usage categories, suggesting specialization in repair of DNA damage types occurring in specific environments. It should be noted that our analysis was confounded by poor representation of some bacterial phyla, noisy data on bacterial physiology, such as conflicting or missing data on oxygen use, optimal growth temperature, and Gram staining. Nonetheless, we believe that our results may serve as a valuable resource for better understanding of the functional roles of DNA polymerases in bacteria. Moreover, these results may guide searches for other, potentially novel, proteins involved in DNA replication and repair.

## Supplementary Material

gkag133_Supplemental_Files

## Data Availability

Structural models and associated metadata are available in Zenodo, at https://doi.org/10.5281/zenodo.18010543. Other data are available in the article and the Supplementary Material.
