# Supplementary material for: Diversity and distribution of bacterial DNA polymerases
Source: Nucleic Acids Res. 2026 Feb 23;54(4):gkag133. doi: 10.1093/nar/gkag133 (PMC12926913; doi:10.1093/nar/gkag133)
Supplement: gkag133_Supplemental_Files [file gkag133_supplemental_files.zip › Bacterial_DNA_polymerases_supplement.pdf]

Diversity and distribution of bacterial DNA polymerases

SUPPLEMENTARY FIGURES AND TABLES

Kęstutis Timinskas, Darius Kazlauskas, Albertas Timinskas and Česlovas Venclovas

Institute of Biotechnology, Life Sciences Center, Vilnius University, Saulėtekio av. 7, Vilnius LT-10257, Lithuania

Corresponding author:

Česlovas Venclovas
Institute of Biotechnology, Life Sciences Center
Vilnius University
Saulėtekio 7,
Vilnius LT-10257, Lithuania

Phone: +370-5-223-4368
Fax: +370-5-223-4367
E-mail: ceslovas.venclovas@bti.vu.lt

Taxonomic groups	% of genomes that have polymerases of specific family					Number of genomes
	C	A	Y	X	B	
Tenericutes	100	41	85	0	0	101
Thermotogae	100	100	32	11	5	19
Firmicutes (Bacilli, Erysipelotrichia)	100	99	98	38	3	346
Firmicutes (Clostridia and other)	100	98	94	28	4	172
Fusobacteria	100	100	94	0	11	18
Actinobacteria	100	99	98	8	2	530
Alphaproteobacteria	100	99	81	2	1	363
Epsilonproteobacteria	100	100	69	7	1	83
Deltaproteobacteria	100	100	92	31	25	72
Beta, Gamma and all other Proteobacteria	100	97	89	6	46	876
Cyanobacteria, Chlamydiae	100	100	22	0	12	50
Deinococcus-Thermus	100	100	57	100	24	21
Aquificae, Thermodesulfobacteria	100	100	7	100	7	15
Planctomycetes, Verrucomicrobia	100	90	100	90	6	52
Acidobacteria, Chloroflexi	100	100	70	63	20	30
Bacteroidetes	100	97	92	31	0	226
Chlorobi	100	100	67	11	100	9
Spirochaetes	100	100	61	0	23	44
All other phyla	100	98	70	42	14	43

Supplementary Figure S1. Distribution of DNA polymerases from different families in the genomes of specific taxonomic groups. Cell color intensity is based on the percentage of members of a particular taxonomic group that have at least one copy of corresponding polymerase.

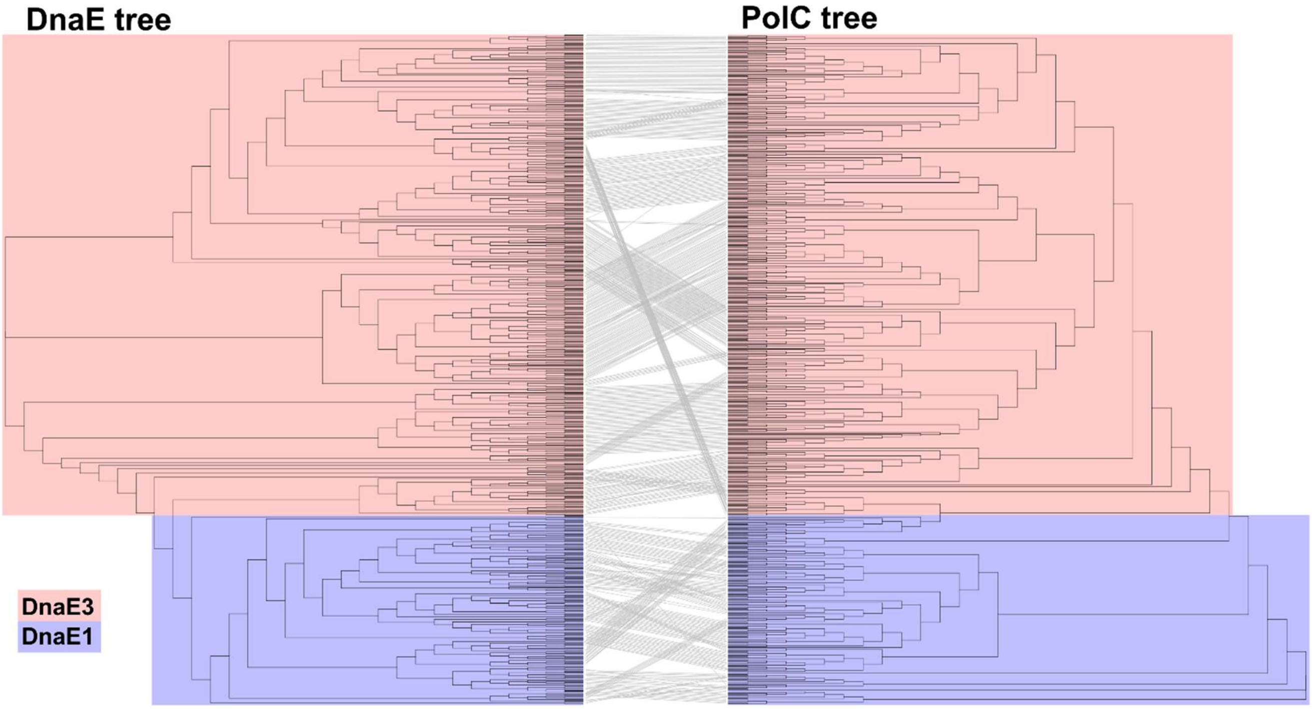

Supplementary Figure S2. Tanglegram of the PolC phylogenetic tree and the tree of DnaE (DnaE1/DnaE3) polymerases co-occurring with PolC. There are no edge crossings between PolC-DnaE1 and PolC-DnaE3 sections of tanglegram.

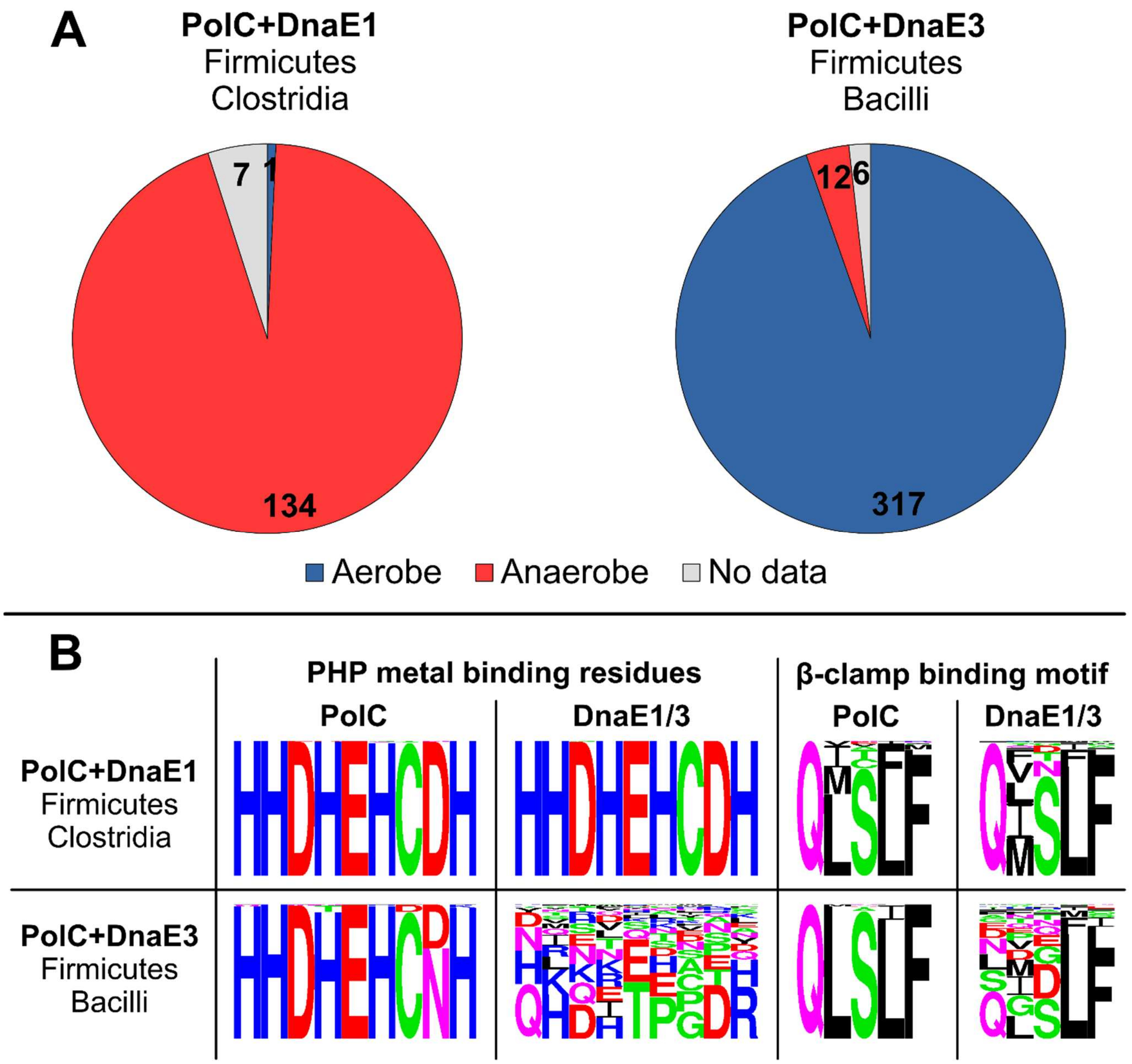

Supplementary Figure S3. Differences between Firmicutes classes Clostridia and Bacilli based on (A) oxygen usage and (B) patterns of the PHP active site (conservation of only the nine metal-binding residues) and the β-clamp binding motif.

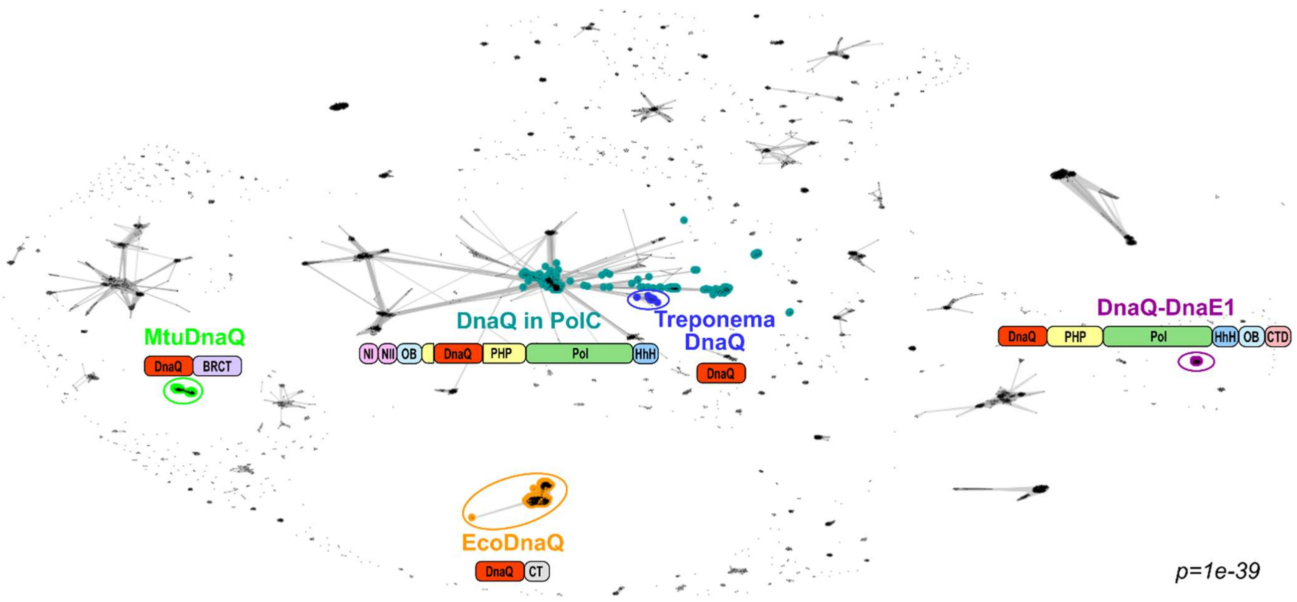

Supplementary Figure S4. CLANS clustering of all DnaQ homologs identified in analyzed proteomes. EcoDnaQ – DnaQ homologs similar to *E. coli* DnaQ; MtuDnaQ – DnaQ homologs similar to *M. tuberculosis* DnaQ; PolC-DnaQ – all DnaQ homologs that are inserted into PolC polymerase; DnaQ-DnaE1 – DnaE1 polymerases with an N-terminal exonuclease domain; Treponema DnaQ – closely related DnaQ homologs that formed good dimer complexes with DnaE1 based on AF3 modeling results (10/11 cases), identified mostly in *Treponema* genus bacteria of Spirochaetes phylum.

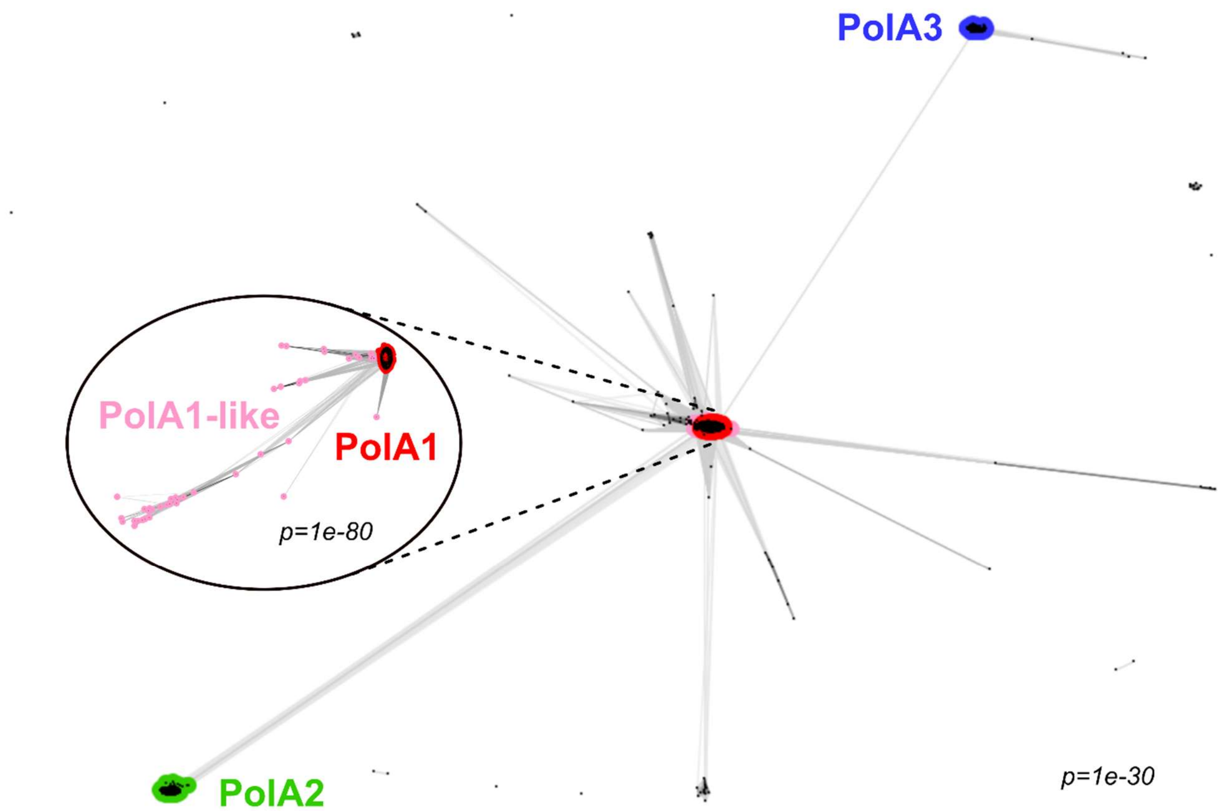

Supplementary Figure S5. Sequence clusters of A-family DNA polymerases. There are three major PolA clusters, PolA1, comprising *E. coli* Pol I orthologs, PolA2 and PolA3. PolA1-like polymerases, lacking the 5′-3′ exonuclease domain, are similar to PolA1 polymerases. Their differences emerge only at a stringent p-value cutoff as shown in the inset.

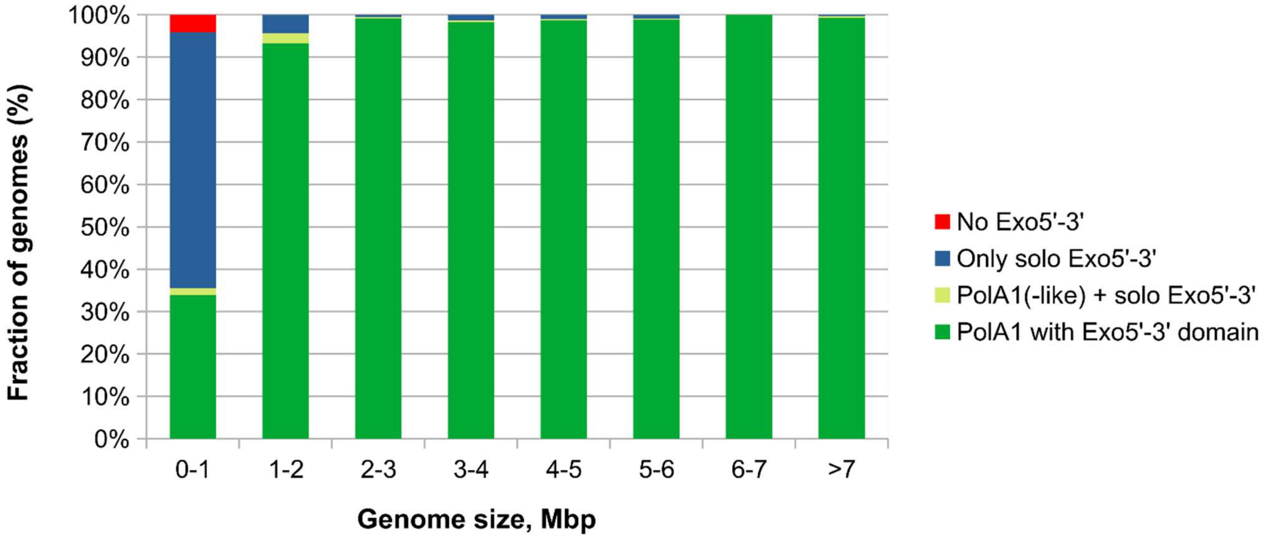

Supplementary Figure S6. Relationship between the genome size and the presence/absence of the 5′-3′ exonuclease domain either as part of PolA or as a standalone protein.

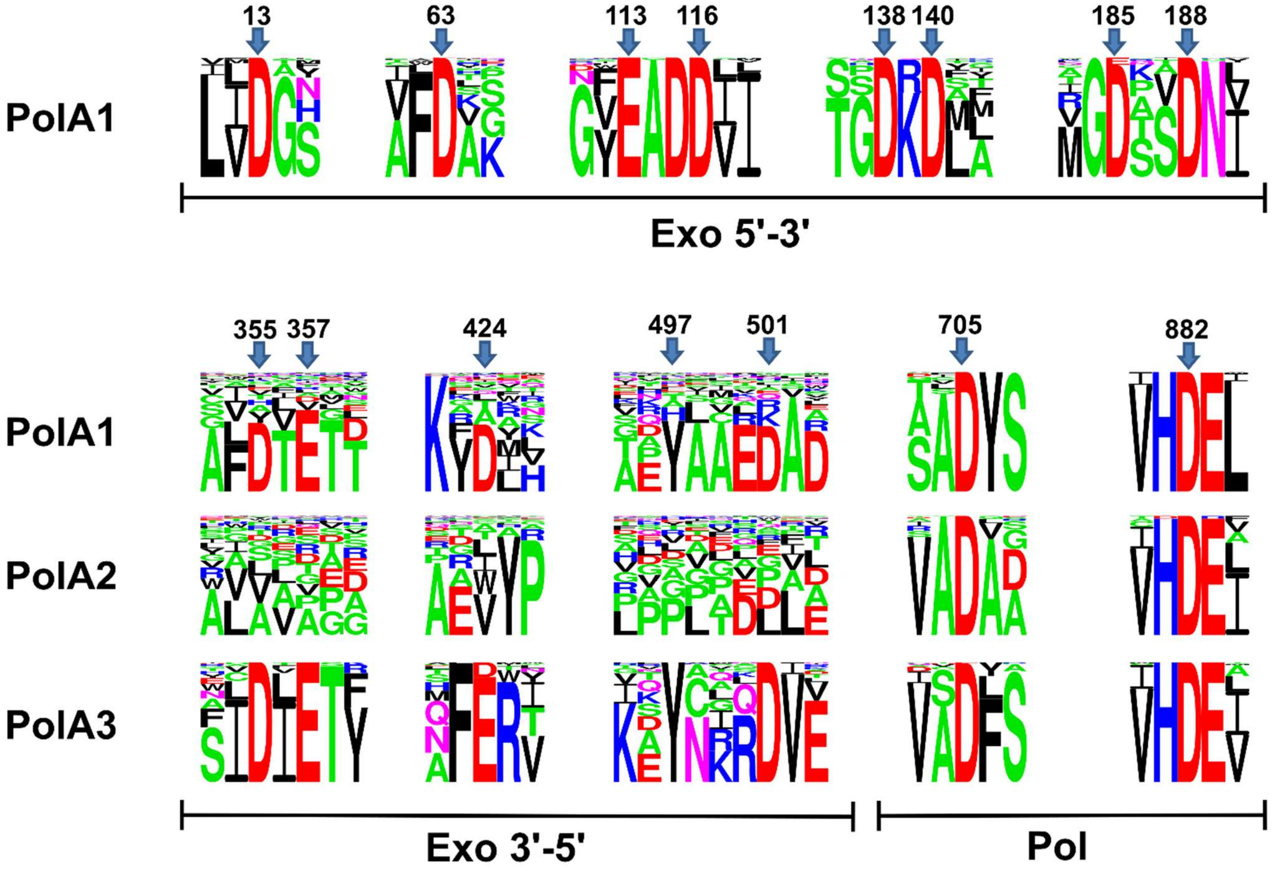

Supplementary Figure S7. Conservation of the active site motifs in the 5'-3' exonuclease, the 3'-5' exonuclease and the polymerase domains for the three major groups of A-family DNA polymerases. The 5'-3' exonuclease domain is present only in PolA1. Numbering corresponds to *E. coli* Pol I sequence (NCBI ID: AAC76861).

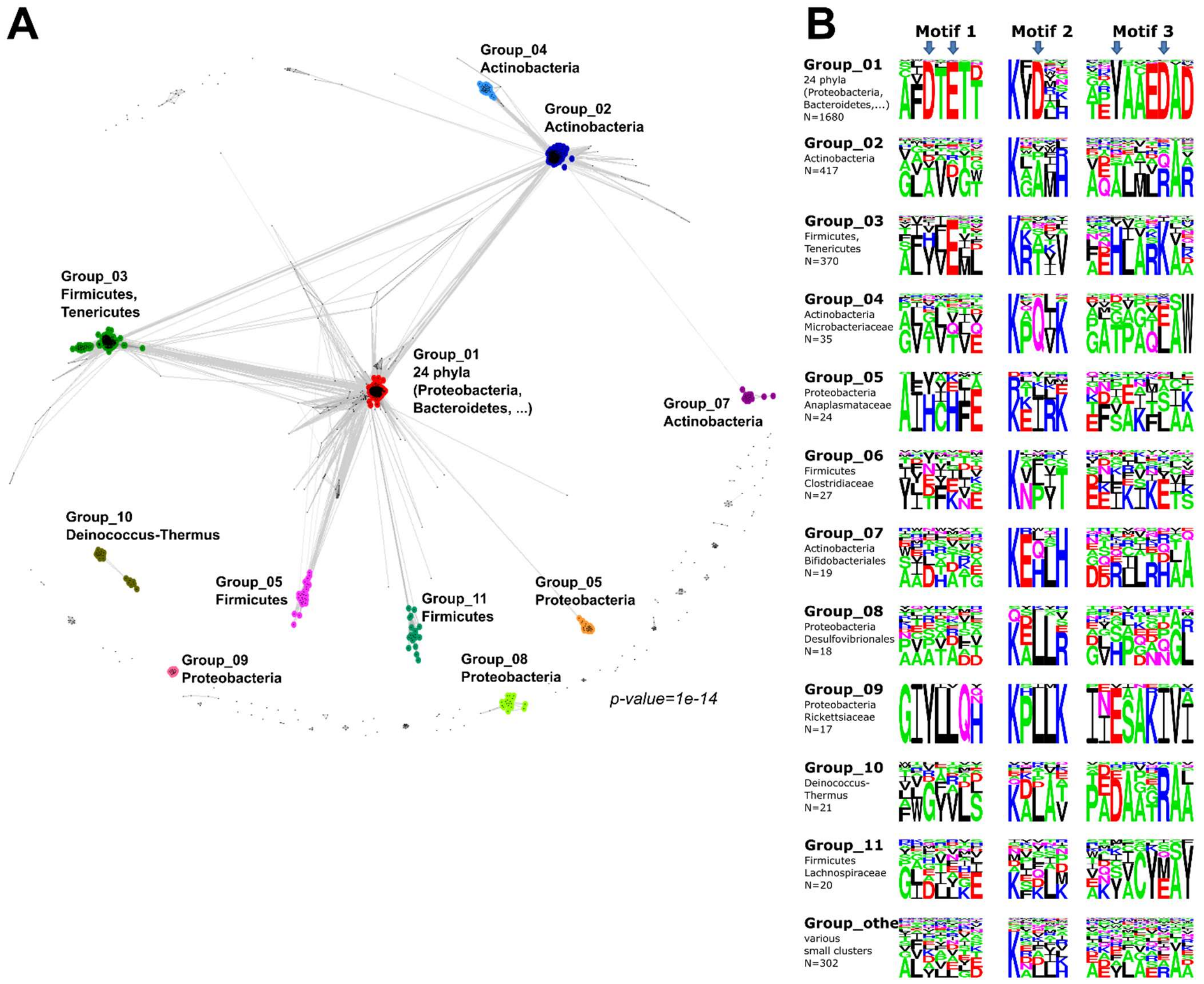

Supplementary Figure S8. Grouping of both catalytically active and inactive 3'-5' exonuclease domains in the PolA1 polymerases. (A) Sequence clusters obtained using CLANS. Group_01 corresponds to the catalytically active 3'-5' exonuclease domains, the remaining groups have an inactive 3'-5' exonuclease domain. (B) Sequence logos of the 3'-5' exonuclease active site motifs for the groups shown in (A).

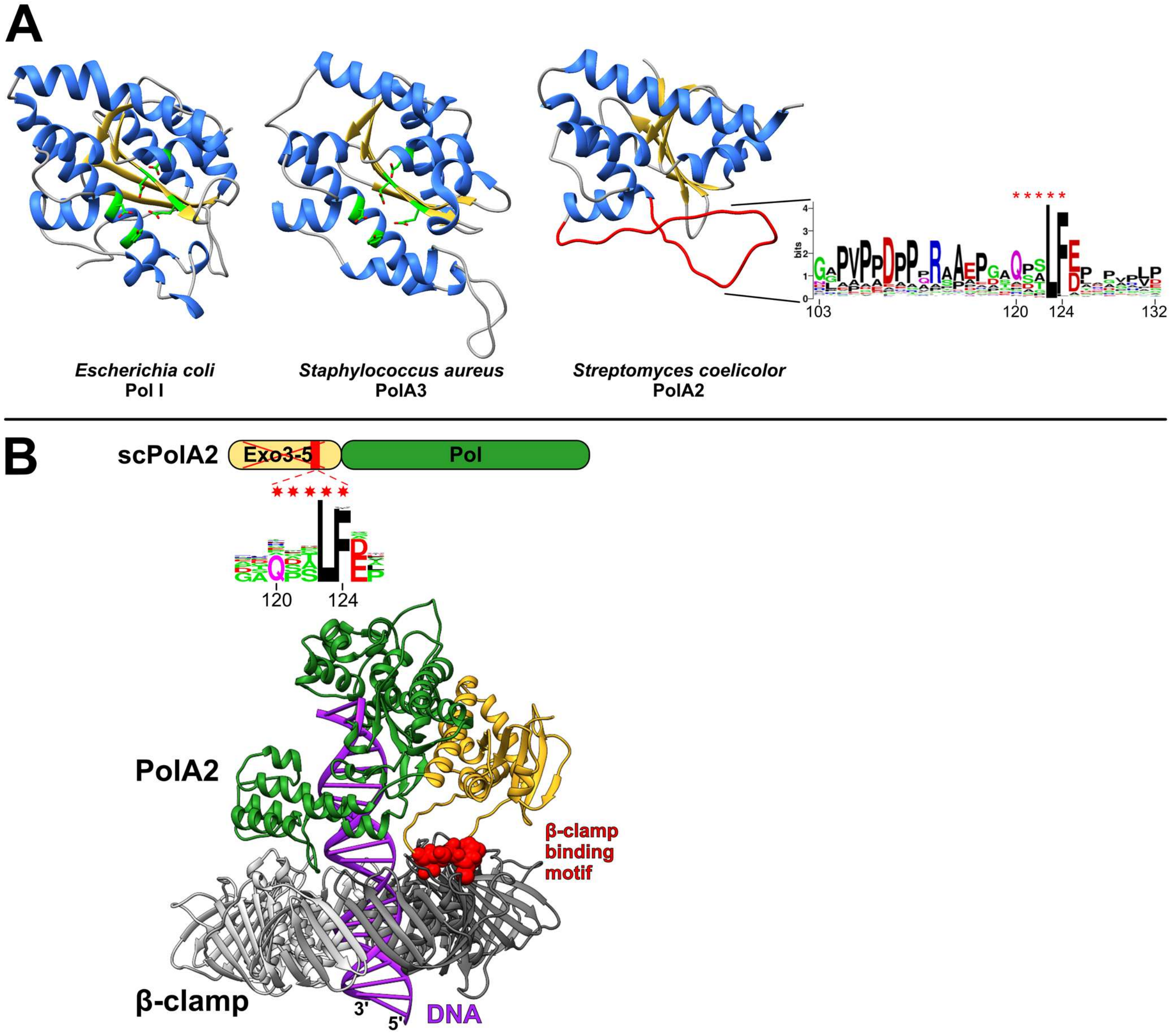

Supplementary Figure S9. PolA2 β-clamp binding motif. (A) Comparison of 3'-5' exonuclease domains of *E. coli* PolA1 (PDB ID: 1KFS), *S. coelicolor* PolA2 (model, AlphaFold database (AFDB) ID: AF-Q8CJW7-F1) and *S. aureus* PolA3 (AFDB ID: AF-Q2FYA1-F1). Exo3'-5' active site is highlighted in green (PolA1, 'DEDDy', PolA3, 'DEEDy'). Corresponding domain in *S. coelicolor* PolA2 shows structural decay, lacks active site residues and features a long loop (colored in red) with a putative β-clamp binding motif. The residue logo represents the β-clamp binding motif derived from the multiple alignment of PolA2 sequences (numbering corresponds to *S. coelicolor* PolA2, NCBI ID: CAD55317). Red stars indicate the β-clamp binding motif. (B) Schematic representation of *S. coelicolor* PolA2 domain architecture (top) and its AlphaFold model bound to DNA and to the β-clamp (NCBI ID: CAB92998) (bottom). In the model, residues involved in binding the β-clamp are shown in red. Model confidence scores: pLDDT = 83, pTM = 0.87, ipTM = 0.85. Additional model details are available in Supplementary data file 4.

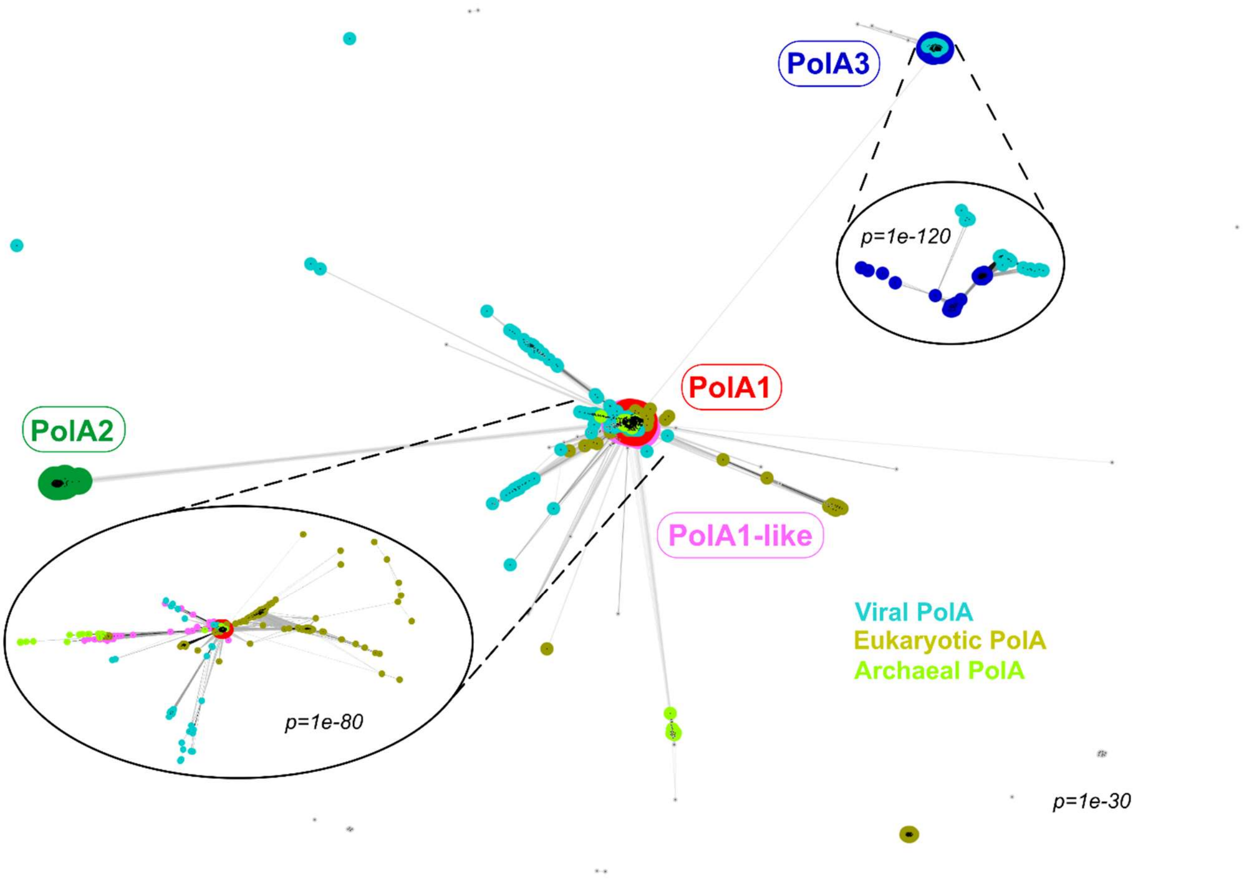

Supplementary Figure S10. Clustering of bacterial PolA DNA polymerases together with eukaryotic, viral and archaeal PolAs. Bacterial PolA3 group forms a joint cluster with phage PolA sequences. The PolA2 cluster does not include any other sequences. PolA3 cluster and the central cluster including bacterial PolA1 and PolA1-like polymerases are also shown zoomed in at different p-value. Eukaryotic, viral and archaeal PolAs were identified by searching appropriate sections of RefSeq (v.2024-12) database with HMMER using PolA1-core, PolA1-like, PolA2 and PolA3 polymerase domain sequence alignments as queries.

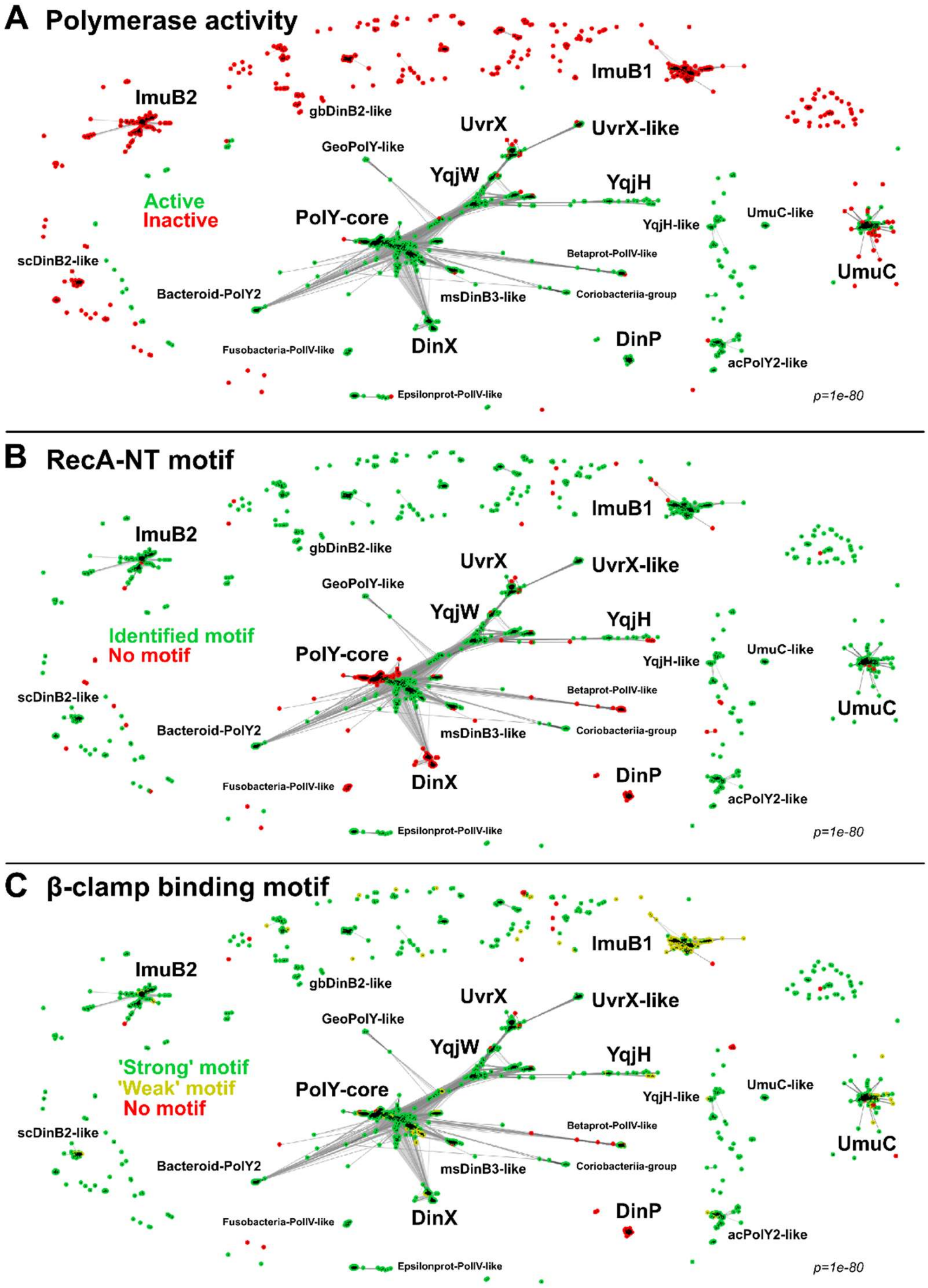

Supplementary Figure S11. Y-family sequence clustering figure (same as in Figure 2C), colored by the presence of (A) polymerase active site (B) RecA-NT motif, and (C) β-clamp binding motif.

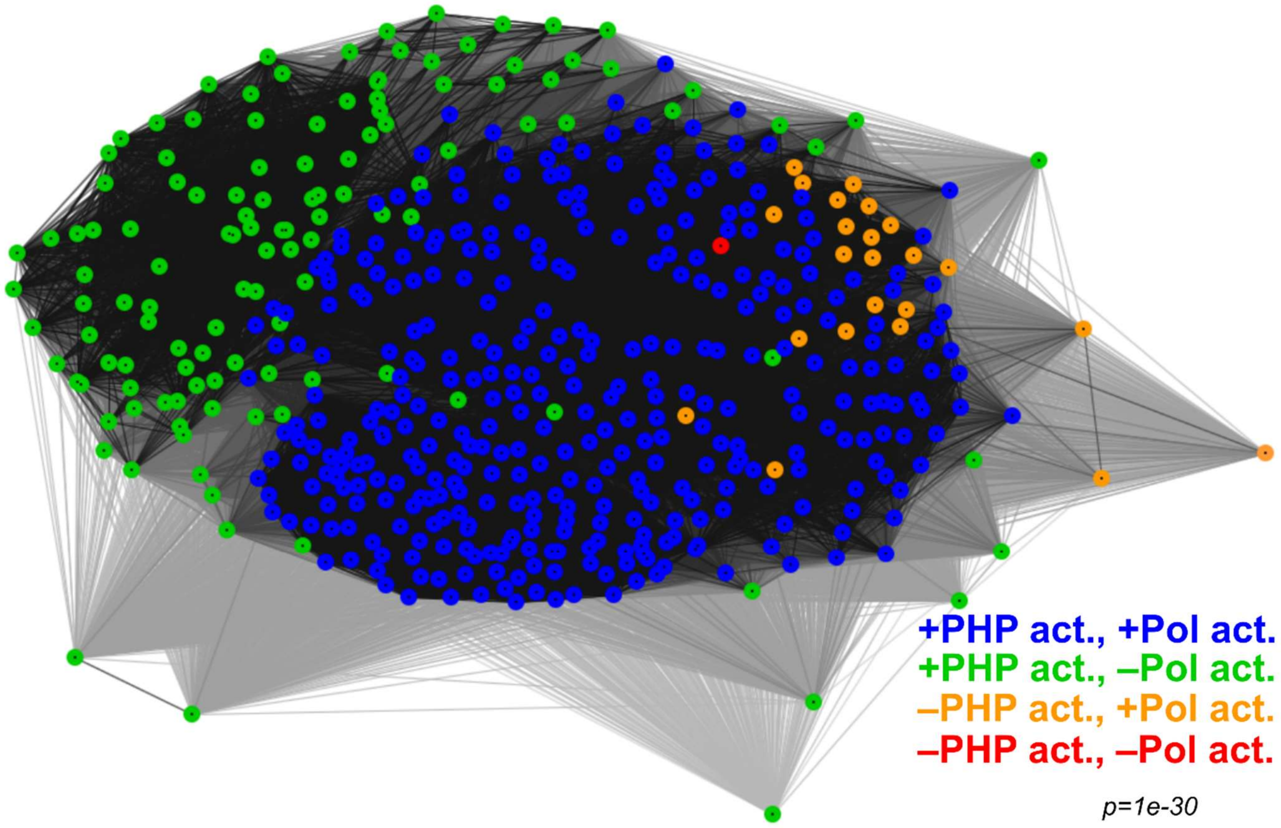

Supplementary Figure S12. Clustering of only PolX1 using CLANS. Colors are based on combination of active (+PHP act.) and inactive (-PHP act.) PHP domains with active (+Pol act.) and inactive (-Pol act.) polymerase domains. Domain activity was assigned based on the presence of the conserved active site motifs: 'HHDHEHHDH' for PHP and 'DDD' for polymerase.

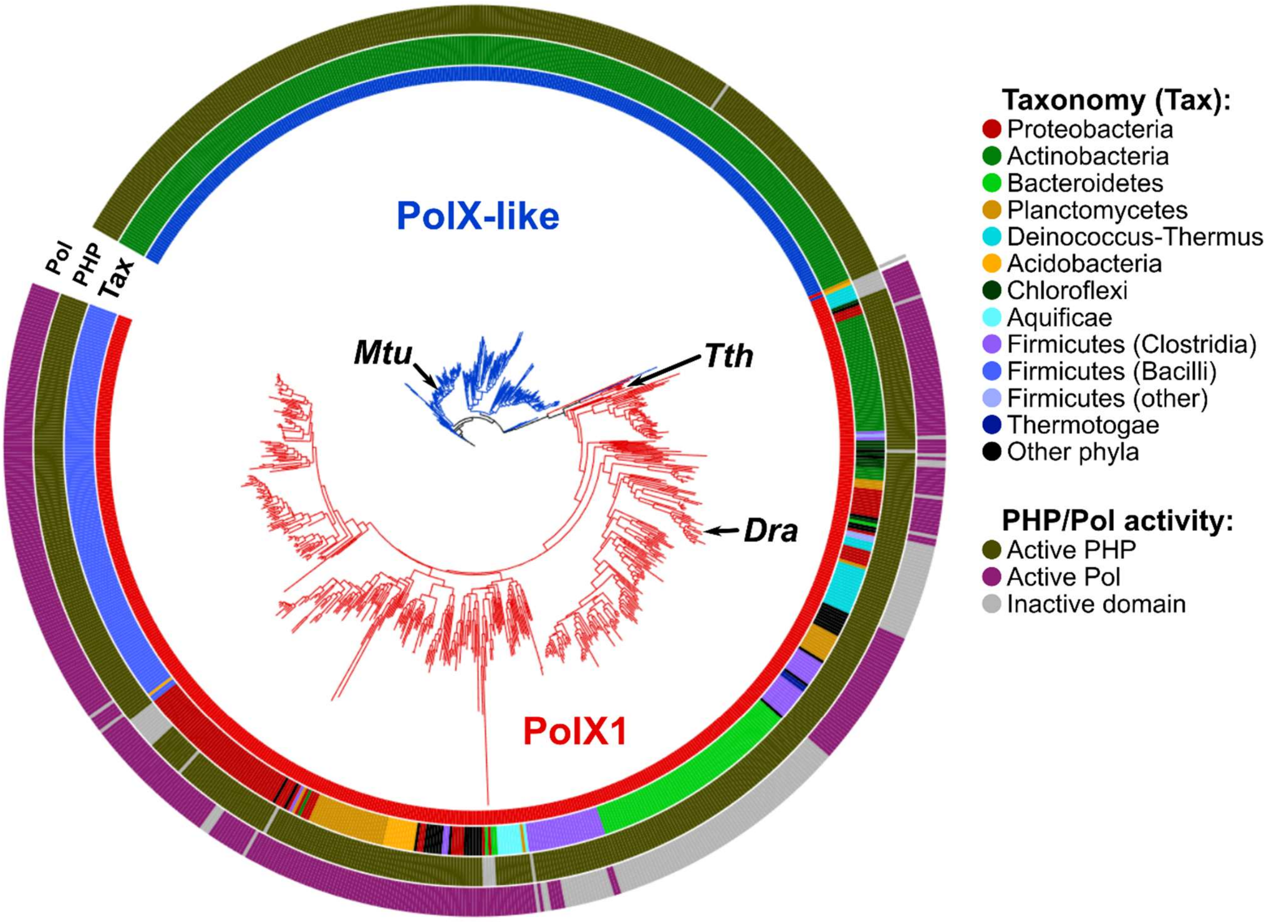

Supplementary Figure S13. Phylogenetic tree of PHP domains from PolX and PolX-like proteins. Tax, Taxonomy, Pol, PolX with the 'DDD' polymerase active site motif; PHP, PHP domain with the 'HHDHEHHDH' motif of metal binding residues. Representative organisms: *Dra*, *Deinococcus radiodurans*, *Tth*, *Thermus thermophilus*, *Mtu*, *Mycobacterium tuberculosis*. The raw phylogenetic tree is available in Supplementary data file 3.

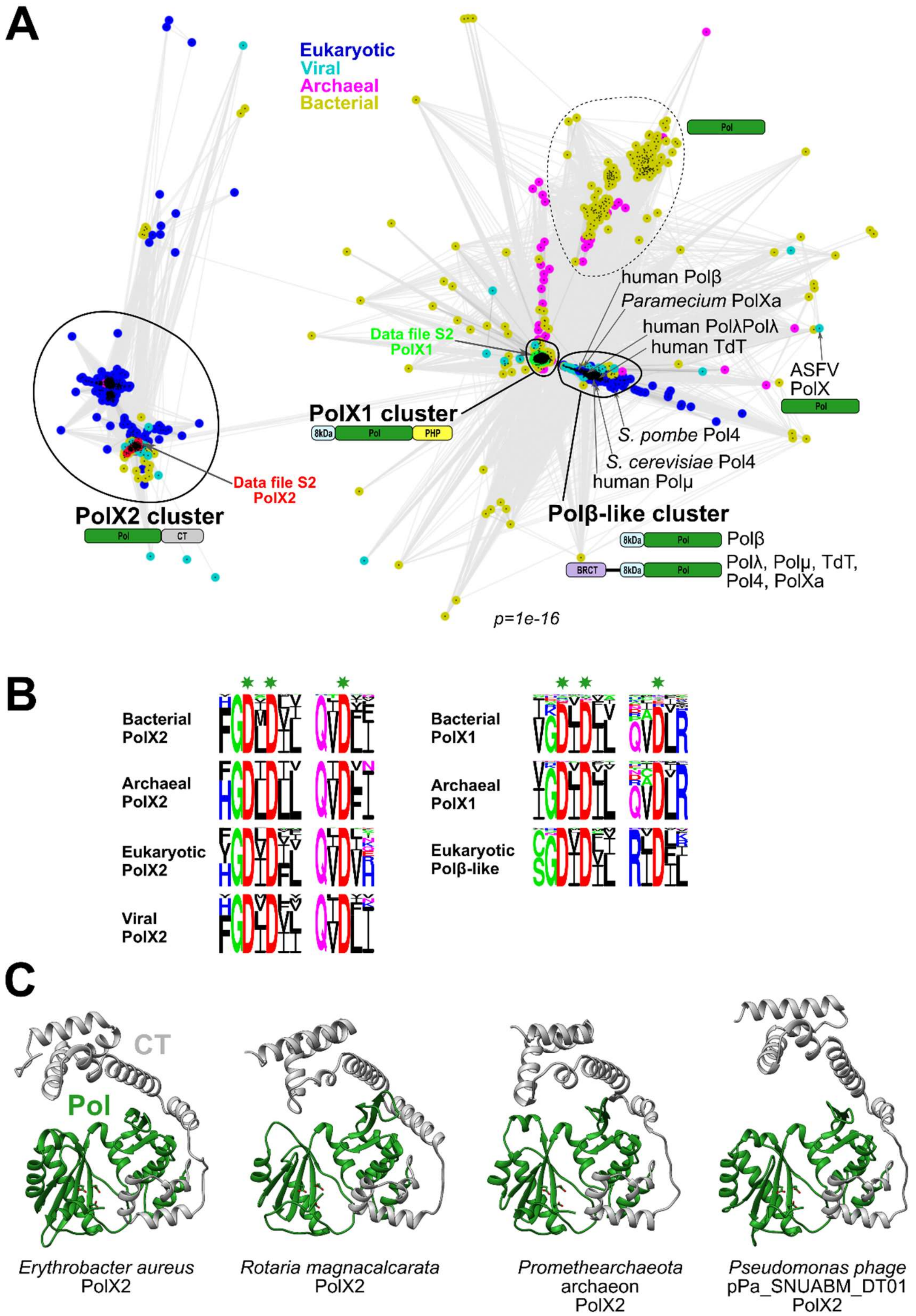

Supplementary Figure S14.

Supplementary Figure S14 (continued). Diversity of PolX homologs. (A) PolX1 and PolX2 sequences from current dataset were clustered using CLANS together with additional homologs identified in ClusteredNR database (1) (queries - PolX1 and PolX2 sequences cut to include only polymerase core; 5 iterations of PSI-BLAST searches; E-value cutoff 1e-5; all results additionally filtered using CD-HIT to maximum 60% sequence identity). The sequences were clustered at p-value cutoff of 1e-25, displayed connections - up to p-value of 1e-16. Additional homologs are colored based on source organism. Typical eukaryotic X family polymerases (Polβ-like cluster) are separated from bacterial PolX1-like polymerases (encircled clusters). Some diverged bacterial PolX most closely related to PolX1 do not have a typical PHP domain (e.g., dashed cicle). PolX2-like cluster is well separated from both typical bacterial PolX1 and eukaryotic PolX. It includes sequences from all domains of life.
(B) Polymerase active site logos of different PolX. Logos were generated for all sequences from the three encircled clusters, separated by taxonomy (current dataset sequences not included). Catalytic aspartate residue positions are indicated by green stars (D197, D199, D234 in *D. radiodurans* PolX, NCBI ID: QEM72418).
(C) Comparison of PolX2 structures from all four domains of life. From left to right: bacterial (model AFDB ID: AF-A0A345YJ29-F1-v4), eukaryotic (AFDB ID: AF-A0A815W160-F1-v4), archaeal (AFDB ID: AF-A0A842RPS6-F1-v4), viral (sequence NCBI ID: QTH80438; model created using AF3, model confidence scores: pLDDT = 90.6, pTM = 0.88).

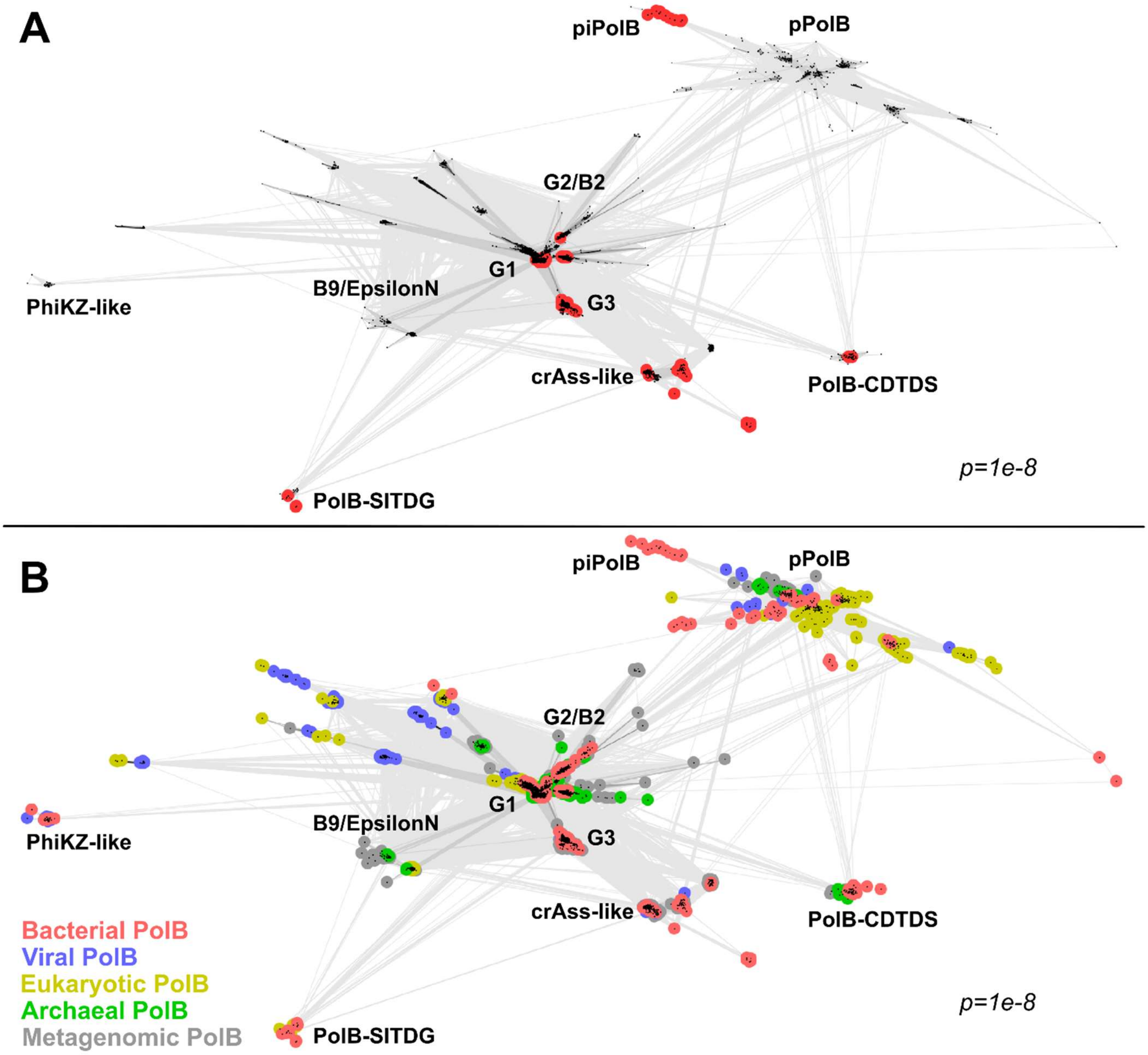

Supplementary Figure S15. Clustering of PolB homologs using CLANS. All B-family DNA polymerases (PolBs) found in the analyzed set of bacterial genomes were clustered with PolBs identified in previous work by Kazlauskas et al. (2). Labels indicate the most abundant PolB groups. (A) Only PolB sequences analyzed in this study are highlighted in red. (B) All PolB sequences are colored by the genomic source.

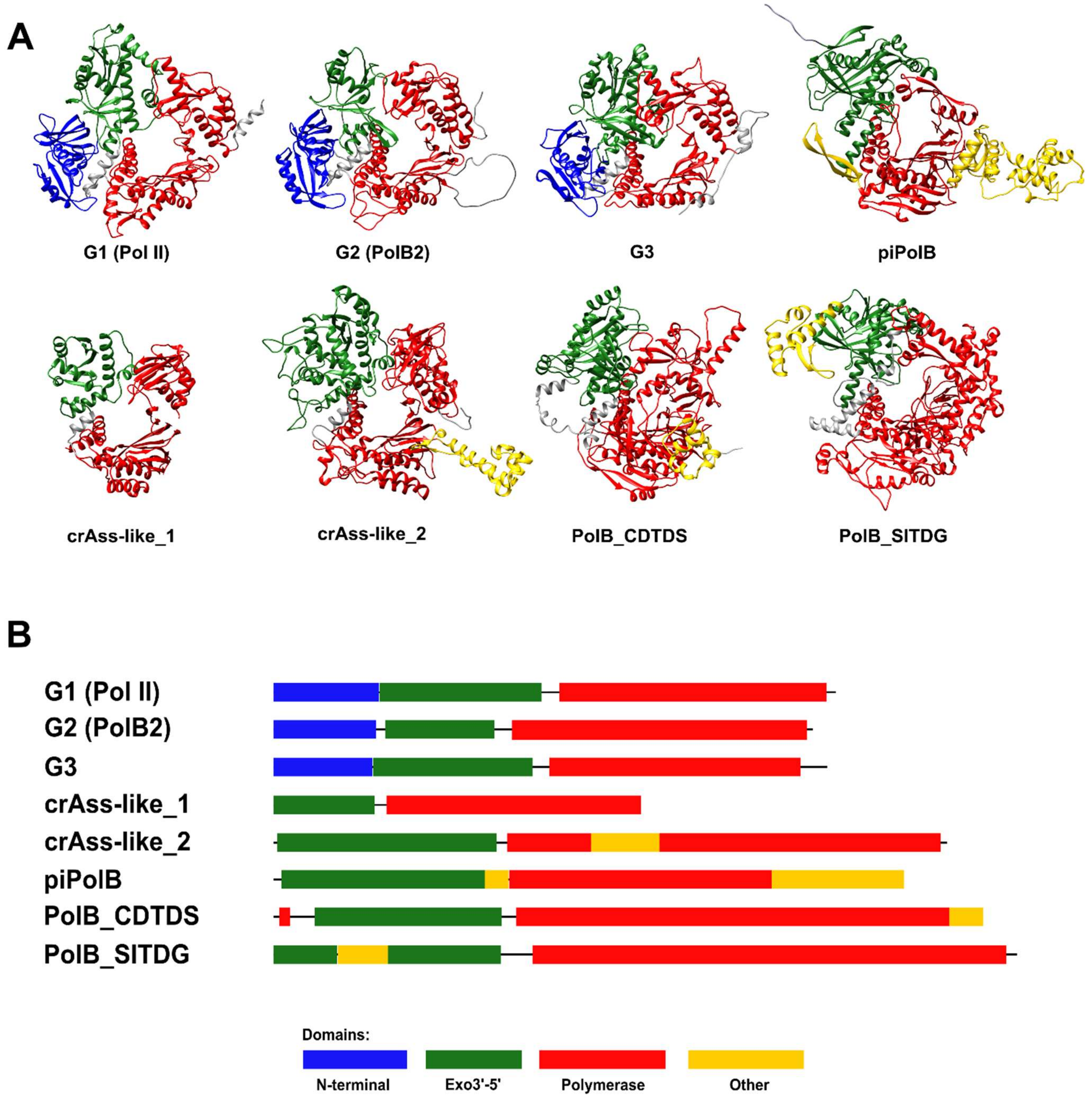

Supplementary Figure S16. Domain architectures for representatives of all identified bacterial B-family polymerase groups. Different colors mark different domains. (A) Structures of PolB representatives (AFDB IDs: G1, AF-A0A6N1C1N4-F1, G2, AF-F2NDD4-F1, G3, AF-D0MIJ2-F1, piPolB, AF-A0A2A4FR66-F1, crAss-like_1, AF-S1QZ24-F1, crAss-like_2, AF-A0A0U3BJH5-F1, PolB_CDTDS, AF-A0A0F7KI06-F1, PolB_SITDG, AF-E0UP04-F1). (B) Schematic representation of the domain architecture of each group, based on structures shown in (A). Length of the schematic corresponds to protein sequence length.

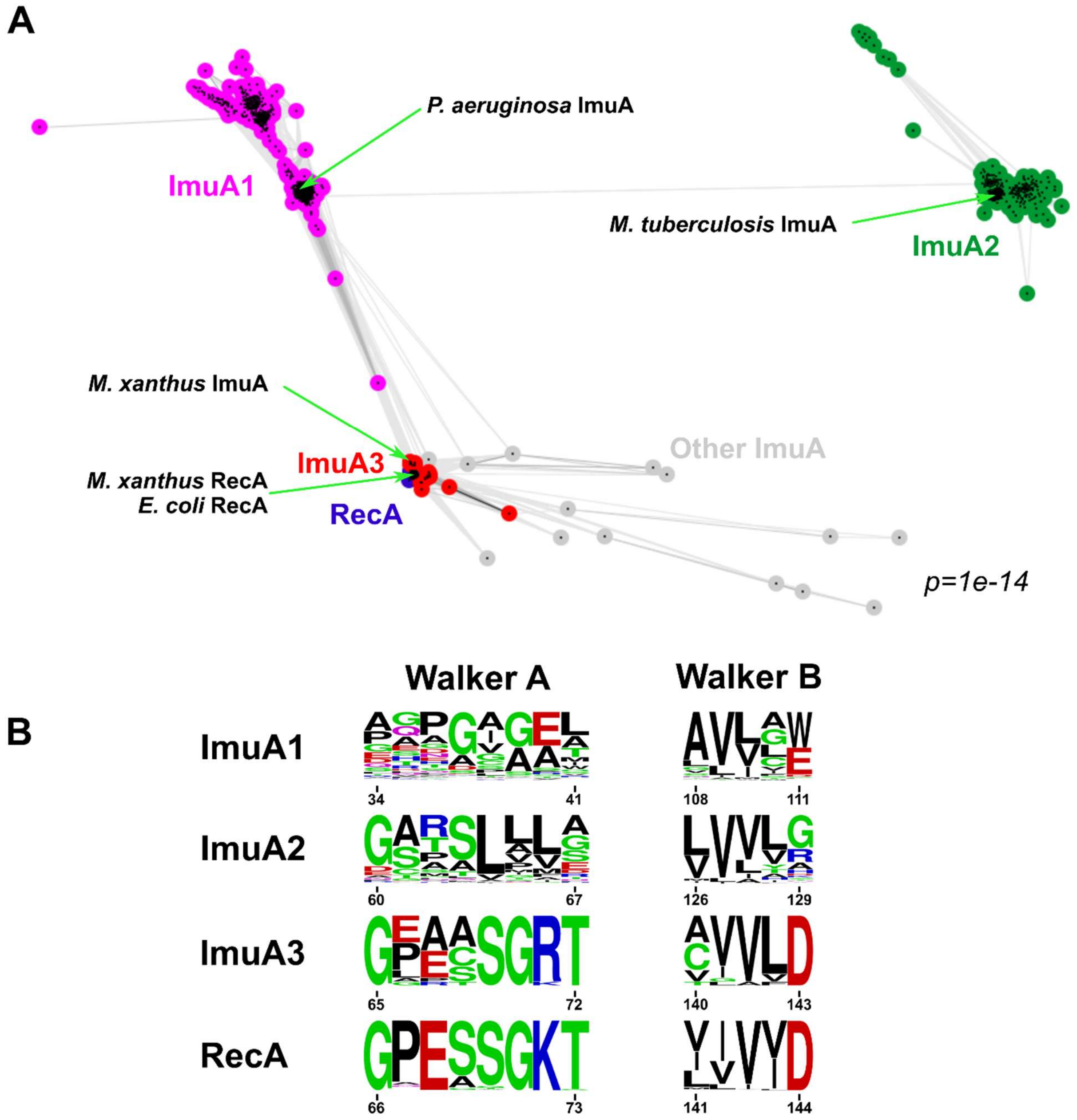

Supplementary Figure S17. Clusters and active site motifs of ImuA homologs. (A) All ImuA assigned to any polymerase, clustered together with a set of RecAs most similar to *E. coli* RecA. Green arrows indicate positions of known representatives for each group. (B) Logos of Walker A and Walker B motifs for the three ImuA groups and RecA. Sequence numbering for each logo is respectively based on *P. aeruginosa* ImuA1 (NCBI ID: AAG04060), *M. tuberculosis* ImuA2 (NCBI ID: CCP46216), *M. xanthus* ImuA3 (NCBI ID: ABF92318) and *E. coli* RecA (PDB ID: 4TWZ).

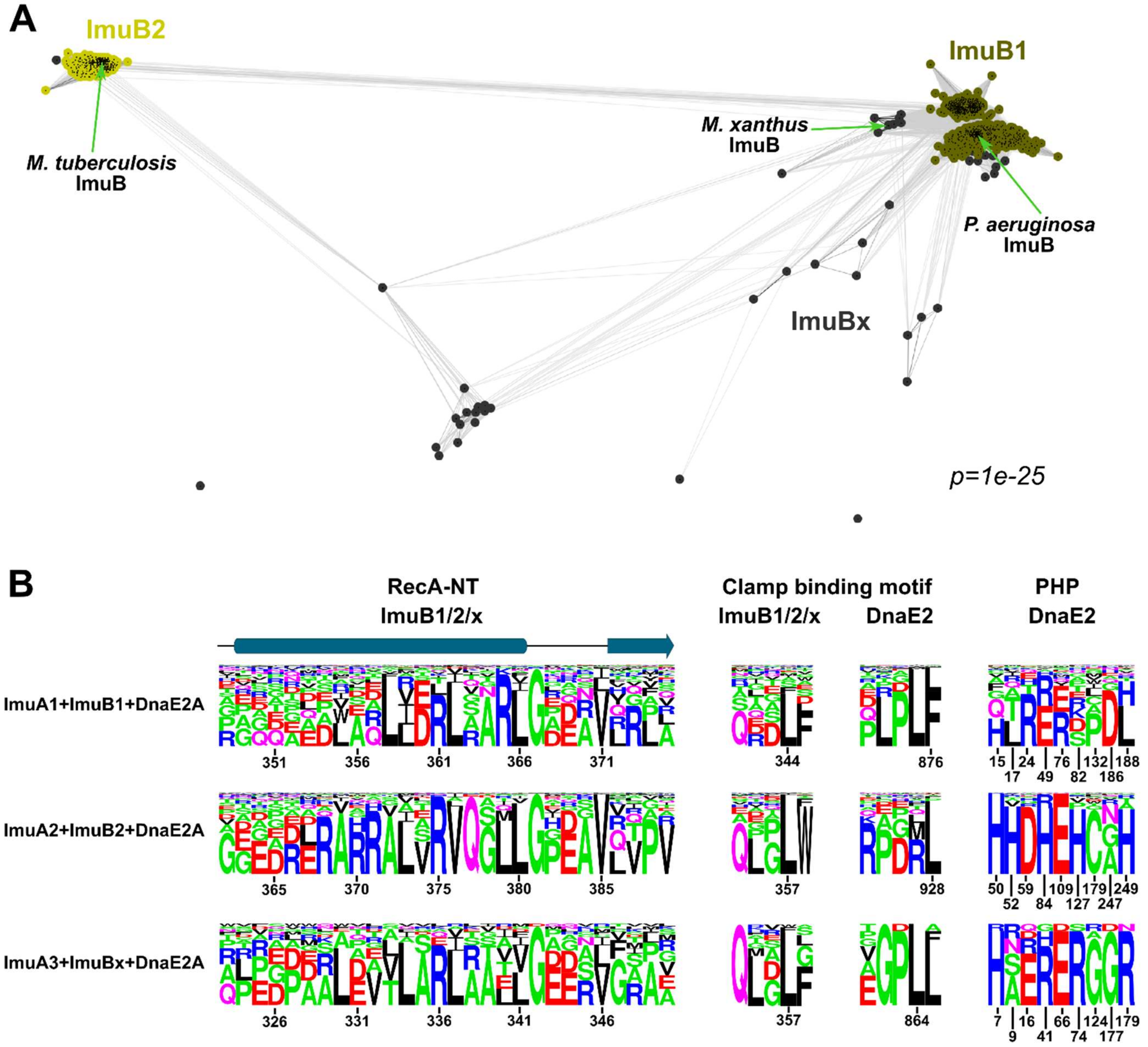

Supplementary Figure S18. Comparison of ImuB sequences and systems. (A) Clustering of ImuB sequences with CLANS. Green arrows indicate positions of dots for representatives of each group. (B) Logos for RecA-NT, clamp binding and PHP active site motifs of ImuB and DnaE2 components of systems that include both different ImuB and ImuA (as noted in description). Sequence numbering for each logo is based on sequences of the three representatives, respectively (NCBI IDs in parentheses): *Pseudomonas aeruginosa* ImuB1 (AAG04059) and DnaE2A (AAG04058), *Mycobacterium tuberculosis* ImuB2 (CCP46215) and DnaE2A (CCP46191), *Myxococcus xanthus* ImuBx (ABF86841) and DnaE2A (ABF91032).

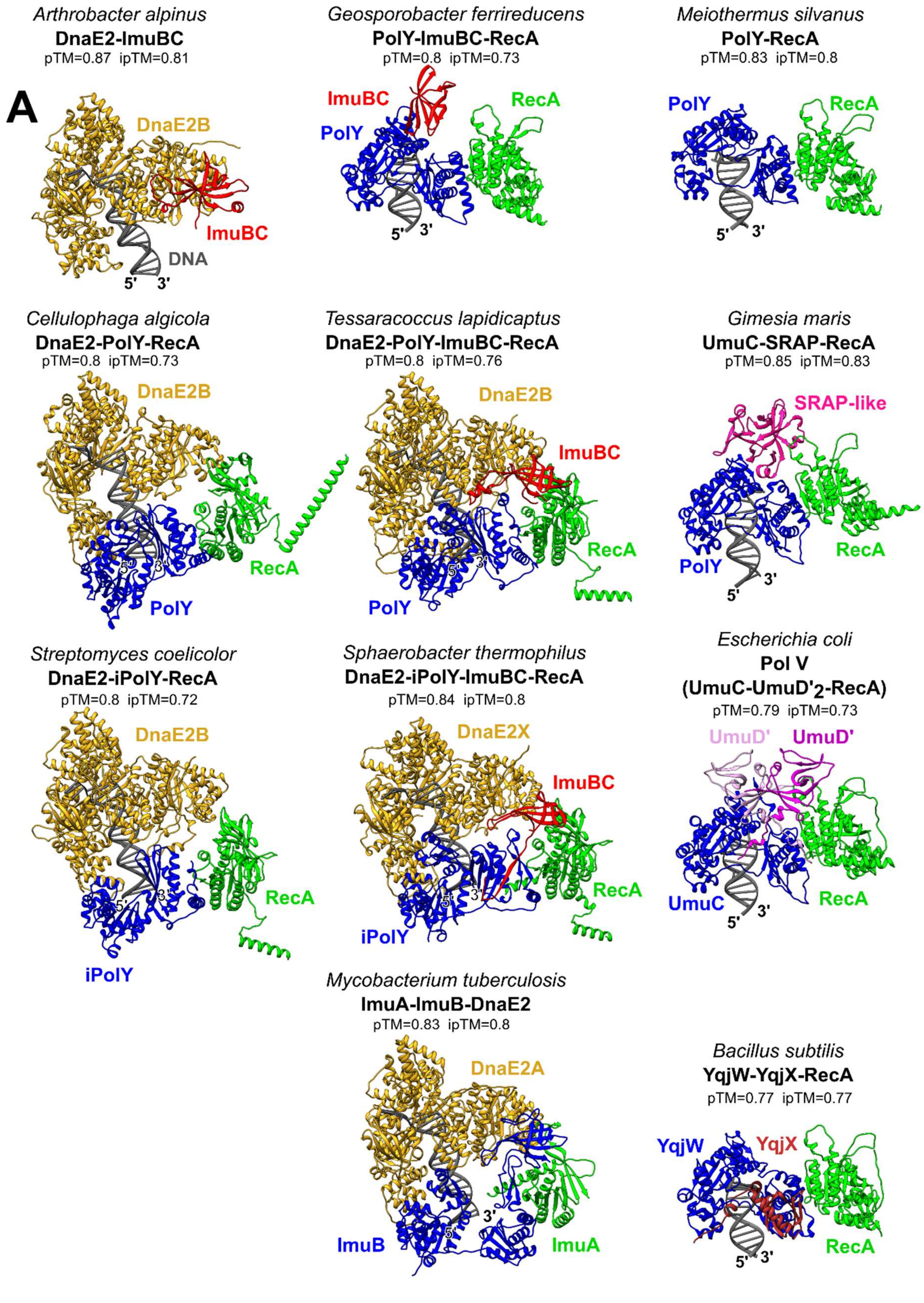

Supplementary Figure S19.

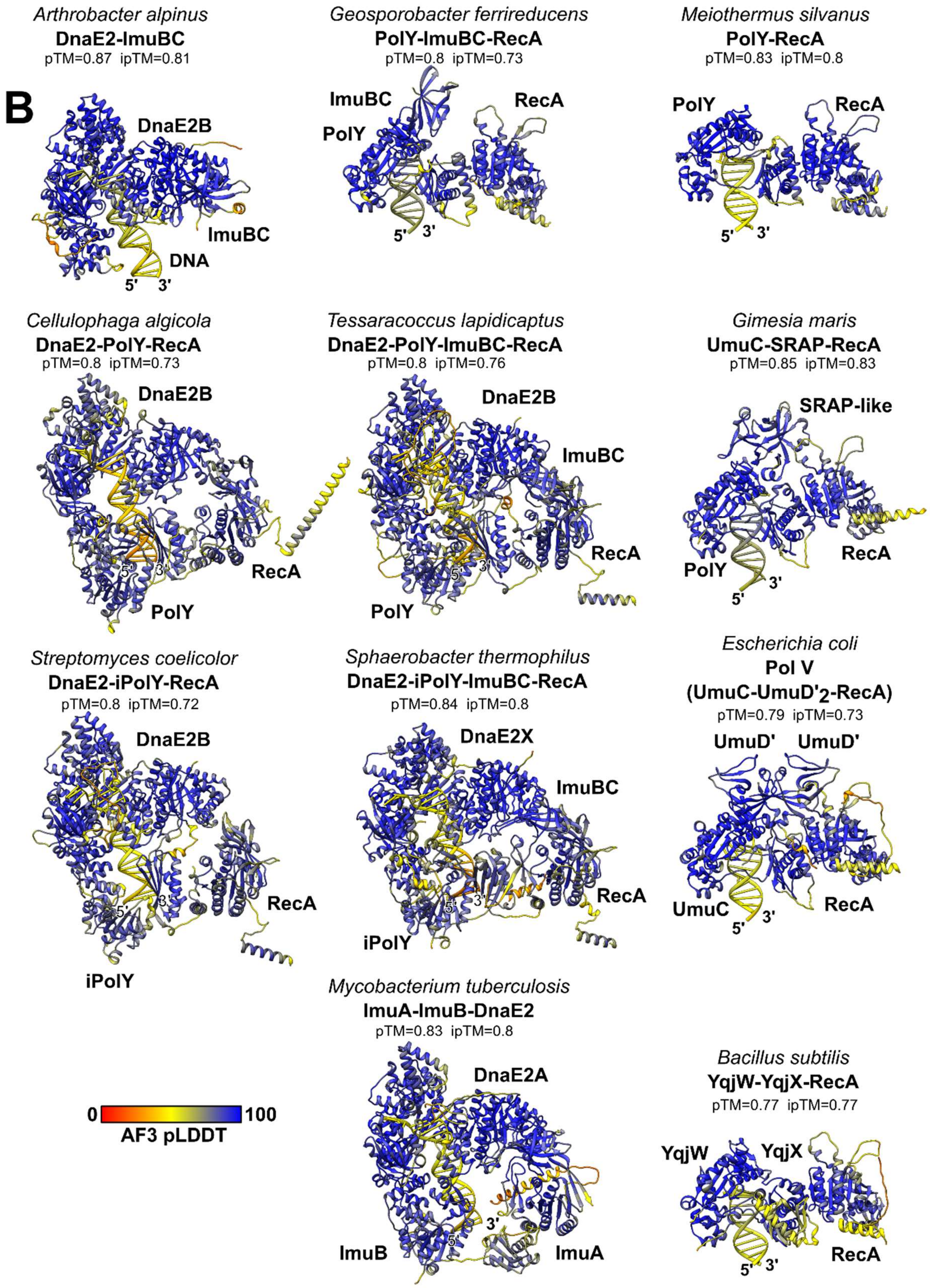

Supplementary Figure S19 (continued).

Supplementary Figure S19 (continued). Representative structural models of different types of known and putative bacterial multimeric error-prone DNA polymerases: (A) models colored by components and (B) local AF3 pLDDT scores. All models were obtained using AlphaFold 3 (AF3), pTM and ipTM quality scores are shown in the figure. Sequence NCBI IDs listed in same order as in system name:
DnaE2-ImuBC: ALV45564, ALV45565;
PolY-ImuBC-RecA: AOT68314, AOT68315, AOT68218;
PolY-RecA: ADH62265, ADH62770;
DnaE2-PolY-RecA: ADV50501, ADV50500, ADV48828;
DnaE2-PolY-ImuBC-RecA: VEP41450, VEP41451, VEP41449, VEP38936;
UmuC-SRAP-RecA: UmuC-like: QDU13704, QDU13703, QDU13483;
DnaE2-iPolY-RecA: CAB50954, CAB50953, CAA15875;
DnaE2-iPolY-ImuBC-RecA: ACZ37666, ACZ39475, ACZ39476, ACZ38361;
Pol V (UmuC-UmuD'-RecA): AAC74268, AAC74267, AAC75741;
ImuA-ImuB-DnaE2: CCP46216, CCP46215, CCP46191;
UvrX-YolD-RecA: CAB14068, CAB14069, CAB13567.
More details for all models are available in Supplementary data file 4.

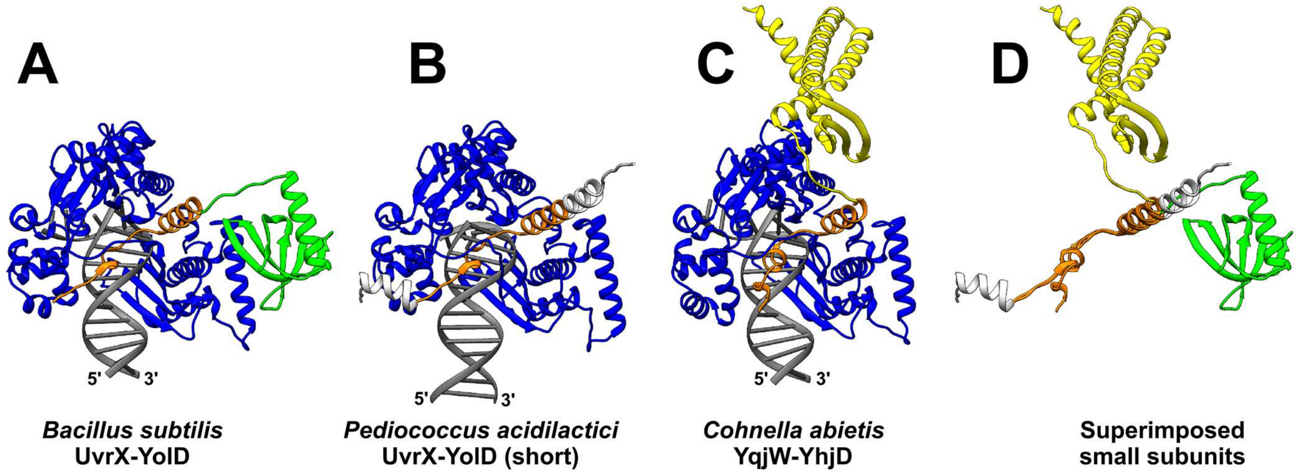

Supplementary Figure S20. Comparison of UvrX/YqjW PolY complexes with different small subunits. (A) Complex with full-length YolD, (B) complex with short YolD variant (without C-terminal domain) and (C) complex with YhjD featuring unrelated C-terminal domain. (D) Superposition of the N-terminal regions of the three types of small subunits (common part colored in orange). All models were obtained using AlphaFold 3. RecA was also included in each model, but here omitted for clarity. Model NCBI IDs in listed complex order (and model AF3 rank scores):

B. subtilis UvrX-YolD-RecA: CAB14068, CAB14069, CAB13567 (0.8);
P. acidilactici UvrX-YolD-RecA: QJW87352, QJW87351, QJW87196 (0.89);
C. abietis YqjW-YhjD-RecA: BBI32855, BBI32856, BBI34359 (0.85).
More details for all models are available in Supplementary data file 4.

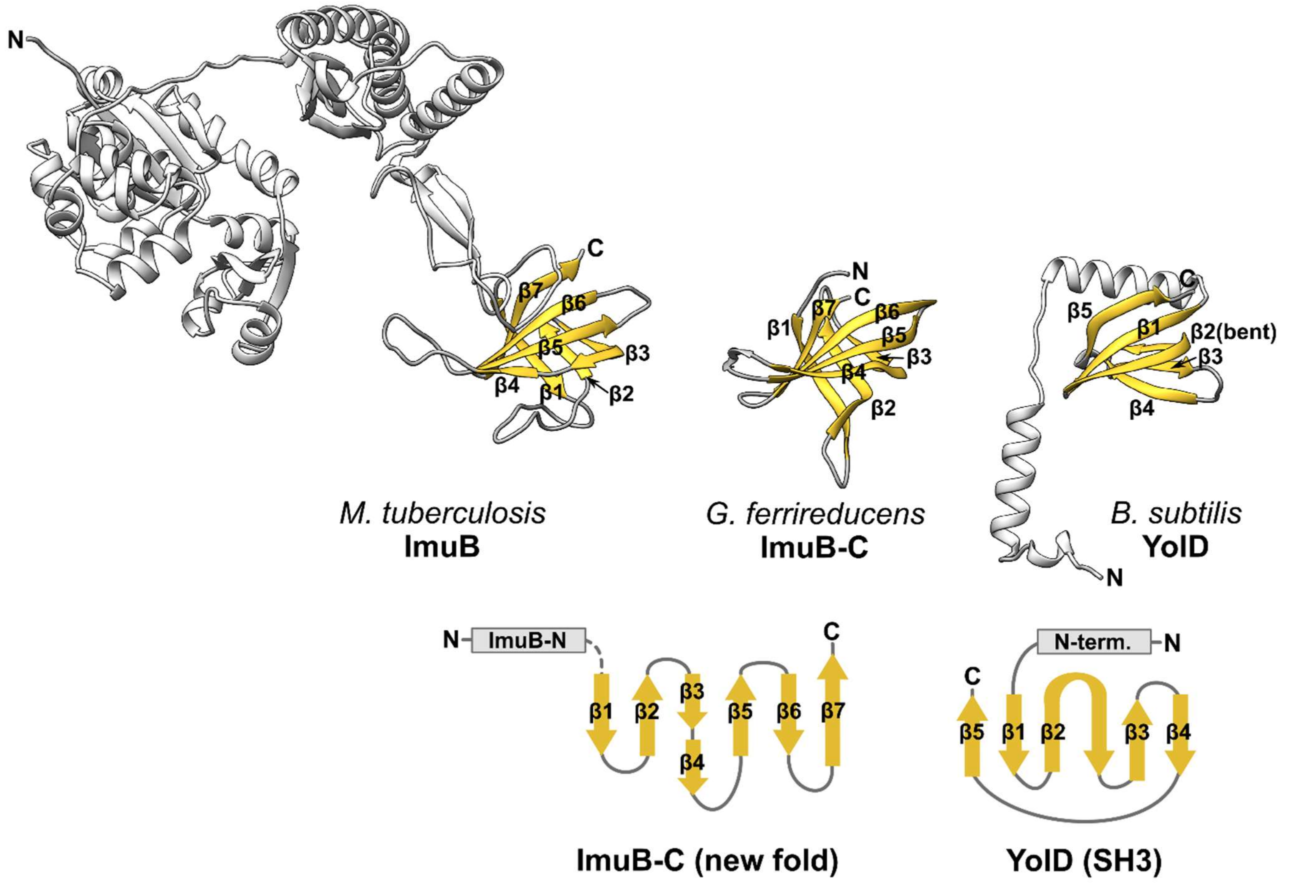

Supplementary Figure S21. Structural comparison of ImuB-C (both as part of full ImuB and as separate protein) and YolD. YolD adopts a typical SH3 fold, while ImuB-C forms a β-barrel of a novel fold. Schematic topology comparison is given below the structures.

ImuB, ImuB-C and YolD structures were taken from each corresponding AF3 model of the full system (Supplementary Figure S18). NCBI sequence IDs: *M. tuberculosis* ImuB - CCP46215, *G. ferrireducen*s ImuB-C - AOT68315, *B. subtilis* YolD – CAB14069.

More details for models are available in Supplementary data file 4.

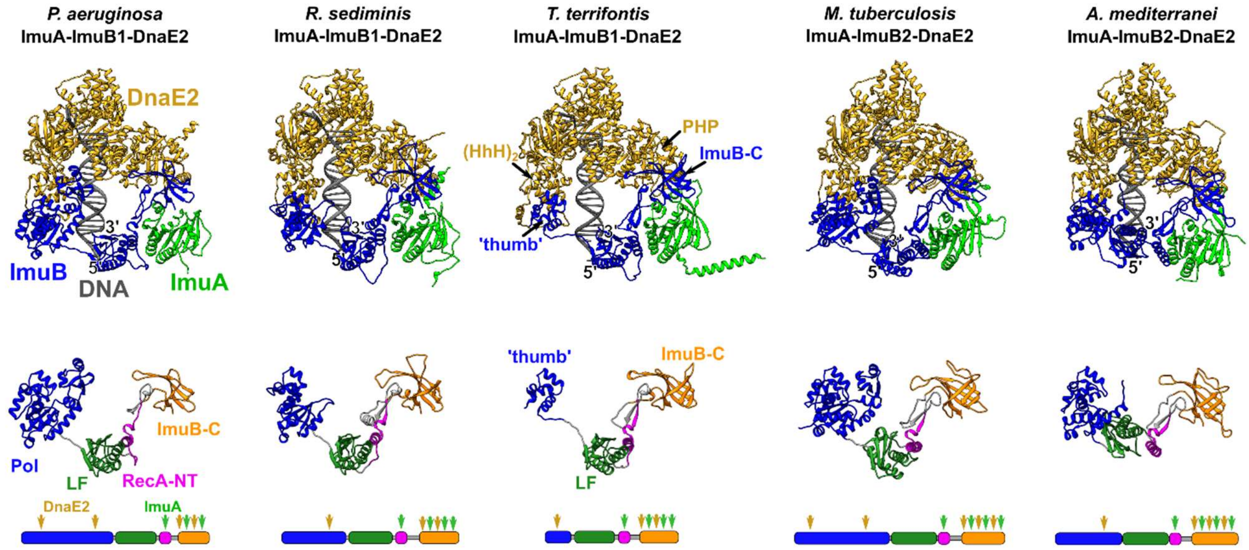

Supplementary Figure S22. Comparison of DnaE2-ImuB-ImuA systems with ImuB in various stages of 'decay' (top), the corresponding ImuB's, colored by domains (middle) and schematic representation of the corresponding sequence (bottom). Colored arrows indicate approximate interaction positions to the two other complex components. Main structural elements mediating the DnaE2–ImuB interaction are indicated with black arrows and colored labels (on the *T. terrifontis* complex).
ImuA, ImuB and DnaE2 NCBI sequence IDs (and AF3 model rank scores):
P. aeruginosa – AAG04060, AAG04059, AAG04058 (0.79);
R. sediminis – QDL37111, QDL37112, QDL37113 (0.81);
T. terrifontis – ASV74860, ASV74858, ASV74857 (0.83);
M. tuberculosis – CCP46216, CCP46215, CCP46191 (0.83);
A. mediterranei – AGT81475, AGT81476, AGT81479 (0.82).
More details for all models are available in Supplementary data file 4.

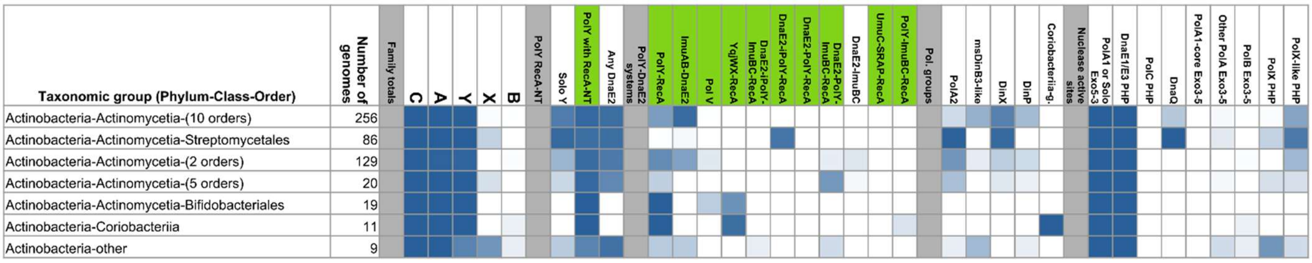

Supplementary Figure S23. Distribution of polymerase families, systems and exonuclease activity between several distinct taxonomic groups of actinobacteria. The blue color intensity of the squares represents percentage of bacteria in the particular group that have at least one copy of the feature.

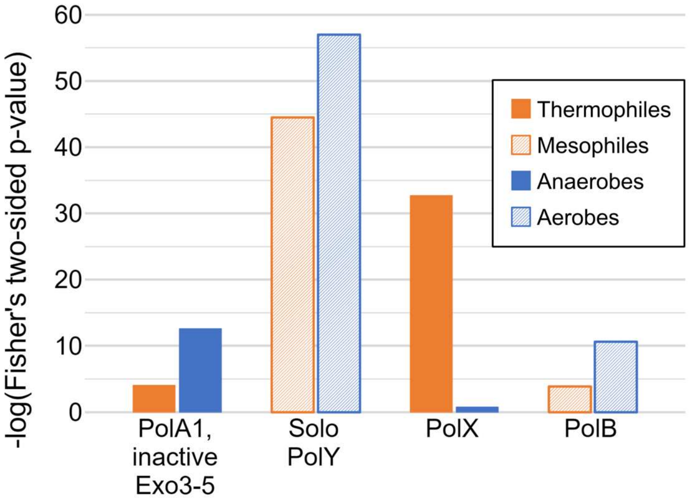

Supplementary Figure S24. Enrichment of DNA polymerase families/groups within two environmental categories, optimal growth temperature and oxygen usage. Y-axis denotes statistical significance of the enrichment.

Supplementary Table S1. Relationship between the presence of PolA1 5′-3′ exonuclease domain and genome properties.

Group	Number of genomes	% of all genomes	Average genome size, Mbp	Average genome GC%
Any PolA1 with Exo5’-3’	2956	96.29	4.14	51.68
Only solo Exo5’-3’	109	3.55	1.48	33.73
No Exo5’-3’	5	0.16	0.27	22.96

Bacterial genomes lacking Exo5’-3’:
Candidatus *Carsonella ruddii* (GCA_000287275.1_ASM28727v1),
endosymbiont of *Pachyrhynchus infernalis* (GCA_004296515.1_ASM429651v1),
Candidatus *Azoamicus ciliaticola* (GCA_902860225.1_Azoamicus_ciliaticola_assembly),
Candidatus *Walczuchella monophlebidarum* (GCA_000709555.1_ASM70955v1),
Candidatus *Portiera aleyrodidarum* (GCA_000292685.1_ASM29268v1).

Supplementary Table S2. Average confidence scores for AlphaFold-Multimer (AFM) structural models of various complexes. Details for all individual models are available in Supplementary data file 4 (first sheet). Error values were calculated as 95% confidence score, based on t-distribution of sample standard deviation.

System	Number of represented species	AFM pLDDT	AFM pTM[a]	AFM ipTM[a]	AFM rank score[a]	VoroMQA score[b]	VoroMQA i_score[b]
**PolV**	5	85±1	0.64 ± 0.04	0.65 ± 0.02	0.65 ± 0.02	0.49 ± 0.01	0.61 ± 0.02
**YqjWX-like**	12	86±1	0.76 ± 0.02	0.74 ± 0.02	0.74 ± 0.02	0.49 ± 0.01	0.52 ± 0.01
**DnaE2X-iPolY-ImuBC-RecA**	5	85±2	0.82 ± 0.04	0.81 ± 0.03	0.81 ± 0.03	0.54 ± 0.02	0.54 ± 0.02
**ImuAB-E2**	10	84±1	0.80 ± 0.01	0.79 ± 0.01	0.79 ± 0.01	0.53 ± 0.01	0.54 ± 0.02
**PolY-DnaE2B-RecA**	5	87±2	0.81 ± 0.02	0.75 ± 0.04	0.76 ± 0.03	0.54 ± 0.01	0.55 ± 0.02
**iPolY-DnaE2B-RecA**	5	86±1	0.80 ± 0.02	0.73 ± 0.05	0.74 ± 0.04	0.52 ± 0.01	0.48 ± 0.05
**PolY-RecA**	5	87±2	0.72 ± 0.06	0.77 ± 0.06	0.76 ± 0.05	0.50 ± 0.02	0.63 ± 0.10
**DnaE2B-PolY-ImuBC-RecA**	5	86±2	0.87 ± 0.10	0.84 ± 0.06	0.85 ± 0.06	0.51 ± 0.01	0.47 ± 0.09
**DnaE2B-ImuBC**	5	88±2	0.87 ± 0.01	0.89 ± 0.05	0.89 ± 0.04	0.52 ± 0.01	0.49 ± 0.03
**UmuC-SRAP-like**	5	88±1	0.85 ± 0.02	0.85 ± 0.05	0.85 ± 0.04	0.52 ± 0.01	0.58 ± 0.06
**PolY-ImuBC-RecA**	5	89±2	0.82 ± 0.05	0.79 ± 0.06	0.79 ± 0.06	0.51 ± 0.01	0.57 ± 0.04

[a]AFM model quality scores: pTM, predicted template modeling score, ipTM, interface predicted modeling score, rank score, AlphaFold score used for model ranking and selection, calculated as 0.8 * ipTM + 0.2 * pTM; ipTM values greater than 0.8 represent confident high-quality predictions, values lower than 0.6 represent unreliable models, and those between 0.6 and 0.8 correspond to a gray zone of model confidence (3).
[b]VoroMQA model accuracy scores: score, global structure accuracy score, i_score, a score of only inter-chain contacts of a complex structure. High accuracy PDB structures typically have scores higher than 0.4 (4).

Supplementary Table S3. Contact areas between small subunit and PolY component of 4 different systems. Contact areas were calculated using VoroContacts (5).

Small subunit	Organism	PolY ID	Small subunit ID	Contact area between PolY and only NT, Å2	Remaining PolY-small subunit contact area, Å2
**(UmuD')₂** 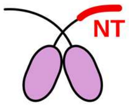	*Escherichia coli*	AAC74268	AAC74267	897	1010
	Salmonella enterica	AAL23540	AAL23541	851	1171
	Acinetobacter pittii	ADY82090	ADY81358	895	1142
**Full YolD** 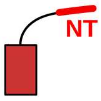	*Bacillus subtilis* (YqjX)	CAB14303	CAB14302	1442	0
	Staphylococcus aureus	ABD30458	ABD31192	1956	24
	Prevotella jejuni	AUI55883	AUI55884	2138	489
**Short YolD** 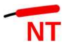	*Pediococcus acidilactici*	QJW87352	QJW87351	1991	0
	Bifidobacterium breve	AHJ17067	AHJ17068	2465	3
	Slackia heliotrinireducens	VEG98733	VEG98732	1698	0
**YhjD** 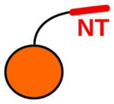	*Cohnella abietis*	BBI32855	BBI32856	1476	1045
	Paenibacillus beijingensis	AJY76487	AJY76488	1523	914
	Cohnella candidum	AYQ71923	AYQ71924	1410	906

Supplementary Table S4. Associations of DNA polymerase families and/or family subsets with environmental factors (optimal growth temperature and oxygen usage). Raw data shows numbers and percentages of analyzed polymerase sets in each environmental category. The statistical data was derived using two-sided Fisher's exact tests and include odds ratios, 95% confidence intervals (CI), and two-sided p-values.

**Polymerase group**	**Temperature**				**Oxygen usage**			
	**Raw data**							
	**In 151 thermophiles**		**In 2704 mesophiles**		**In 2339 aerobes**		**In 479 anaerobes**	
PolA1 with inactive 3′-5′ exonuclease	90 (59.6%)		1167 (43.2%)		968 (41.4%)		286 (59.7%)	
PolA1 with active 3′-5′ exonuclease	55 (36.4%)		1460 (54.0%)		1293 (55.3%)		185 (38.6%)	
Any PolY	96 (63.6%)		2491 (92.1%)		2130 (91.1%)		412 (86.0%)	
Solo PolY	9 (6.0%)		1662 (61.5%)		1526 (65.2%)		123 (25.7%)	
PolY with RecA-NT	94 (62.3%)		2177 (80.5%)		1881 (80.4%)		347 (72.4%)	
Any PolX	88 (58.3%)		384 (14.2%)		364 (15.6%)		87 (18.2%)	
Any PolB	10 (6.6%)		481 (17.8%)		445 (19.0%)		35 (7.3%)	
	**Statistical evaluation**							
	**Enriched in**	**Odds ratio**	**95% CI**	**p-value**	**Enriched in**	**Odds ratio**	**95% CI**	**p-value**
PolA1 with inactive 3′-5′ exonuclease	Thermophiles	1.94	[1.39, 2.71]	9.61e-05	Anaerobes	2.10	[1.72, 2.56]	2.65E-13
PolA1 with active 3′-5′ exonuclease	Mesophiles	2.05	[1.46, 2.88]	3.49e-05	Aerobes	1.96	[1.61, 2.4]	2.83E-11
Any PolY	Mesophiles	6.70	[4.68, 9.6]	4.72e-21	Aerobes	1.66	[1.23, 2.23]	0.001
Solo PolY	Mesophiles	25.17	[12.8, 49.6]	3.20e-45	Aerobes	5.43	[4.35, 6.78]	1.03E-57
PolY with RecA-NT	Mesophiles	2.51	[1.78, 3.53]	4.32e-07	Aerobes	1.56	[1.25, 1.96]	1.62E-04
Any PolX	Thermophiles	8.48	[5.99, 11.86]	2.12e-33	Anaerobes	1.20	[0.935, 1.56]	0.171
Any PolB	Mesophiles	3.05	[1.59, 5.84]	1.43e-04	Aerobes	2.98	[2.08, 4.27]	2.42E-11

REFERENCES

1. https://ncbiinsights.ncbi.nlm.nih.gov/2025/05/22/faster-better-results-protein-blast
2. Kazlauskas, D., Krupovic, M., Guglielmini, J., Forterre, P. and Venclovas, Č. (2020) Diversity and evolution of B-family DNA polymerases. *Nucleic Acids Res*, **48**, 10142-10156.
3. Evans, R., O'Neill, M., Pritzel, A., Antropova, N., Senior, A., Green, T., Žídek, A., Bates, R., Blackwell, S., Yim, J. *et al.* (2021) Protein complex prediction with AlphaFold-Multimer. *bioRxiv*, 2021.2010.2004.463034.
4. Olechnovič, K. and Venclovas, Č. (2019) VoroMQA web server for assessing three-dimensional structures of proteins and protein complexes. *Nucleic Acids Res*, **47**, W437-W442.
5. Olechnovič, K. and Venclovas, Č. (2021) VoroContacts: a tool for the analysis of interatomic contacts in macromolecular structures. *Bioinformatics*, **37**, 4873–4875.
